# Identification of ovarian cancer associated genes using an integrated approach in a Boolean framework

**DOI:** 10.1186/1752-0509-7-12

**Published:** 2013-02-06

**Authors:** Gaurav Kumar, Edmond J Breen, Shoba Ranganathan

**Affiliations:** 1 ARC Centre of Excellence in Bioinformatics and Department of Chemistry and Biomolecular Sciences, Macquarie University, Sydney, NSW, 2109, Australia; 2 Center for Biomarker Research and Personalized Medicine, School of Pharmacy, Virginia Commonwealth University, 1112 East Clay, P.O. Box 980533, Richmond, VA, 23298, USA; 3 Australian Proteome Analysis Facility (APAF), Macquarie University, Sydney, NSW, 2109, Australia; 4 Department of Biochemistry, Yong Loo Lin School of Medicine, National University of Singapore, 8 Medical Drive, Singapore, 117597, Singapore

## Abstract

**Background:**

Cancer is a complex disease where molecular mechanism remains elusive. A systems approach is needed to integrate diverse biological information for the prognosis and therapy risk assessment using mechanistic approach to understand gene interactions in pathways and networks and functional attributes to unravel the biological behaviour of tumors.

**Results:**

We weighted the functional attributes based on various functional properties observed between cancerous and non-cancerous genes reported from literature. This weighing schema was then encoded in a Boolean logic framework to rank differentially expressed genes. We have identified 17 genes to be differentially expressed from a total of 11,173 genes, where ten genes are reported to be down-regulated *via* epigenetic inactivation and seven genes are up-regulated. Here, we report that the overexpressed genes *IRAK1*, *CHEK1* and *BUB1* may play an important role in ovarian cancer. We also show that these 17 genes can be used to form an ovarian cancer signature, to distinguish normal from ovarian cancer subjects and that the set of three genes, *CHEK1, AR,* and *LYN*, can be used to classify good and poor prognostic tumors.

**Conclusion:**

We provided a workflow using a Boolean logic schema for the identification of differentially expressed genes by integrating diverse biological information. This integrated approach resulted in the identification of genes as potential biomarkers in ovarian cancer.

## Background

The development of gene expression microarrays more than a decade ago has led to the study of changes in the mRNA transcripts in disease-related tissues. These transcriptomic analyses from microarrays experiments served as the proxy for protein expression, and thereby revealed important properties of gene sets related to tissue-specificity [1,2]. It has also facilitated the understanding of living cells at a systemic level by linking molecules to biological functions and thus bridging the genotype-to-phenotype gap *via* understanding the organisation of biological pathways [3] and the network of protein interactions [4]. In a seminal review, Hanahan and Weinberg [5] introduced six “*hallmarks of cancer*” (i.e. self-sufficient in growth signals, insensitivity to growth inhibition, evading apoptosis, tissue invasion and metastasis, sustained angiogenesis and limitless replicative potential), while a seventh hallmark (*stemness*) of cancer was concluded through gene expression analysis [6,7]. The remarkable progress in cancer research suggests that hallmarks for cancer need to be extended further by including reprogramming of cellular metabolism to support neoplastic proliferation, acquired cellular properties to avoid immune destruction and genomic instability [8]. In recent years, researchers have made an effort to provide their microarray experiments for further studies through freely available public repositories such as Gene Expression Omnibus (GEO) [9] and ArrayExpress [10].

The knowledge acquired over the years of research suggests that the cancer cells harbour genetic defects that alter the balance of cell proliferation and cell death [11]. This has led to the compilation of a cancer gene list, which has increased steadily over the last two decades. This disease is also highly variable with multiple heterogeneous genetic and epigenetic changes which makes it ideal to study cancer by integrating data from multiple experiments to understand its causes at the cellular level. Therefore, the identification and characterisation of susceptible genes associated with cancer is one of the greatest challenges in today’s biological and medical research. This challenge is partly due to the limitation of statistical methods on which a hypothesis about the value of a statistical parameter is made for the detection of genes effects and their interactions, as multiple biological components work in a concerted fashion. Moreover, biological systems are highly enriched with examples of combinatorial regulation and influence as molecules in signalling pathway and gene regulatory pathway jointly affect the cellular state [12]. In order to explore the combinatorial influence of multiple factors, Boolean-based logic is a popular approach for SNP association studies [13,14] and in cancer [12,15,16].

**Figure 1:**
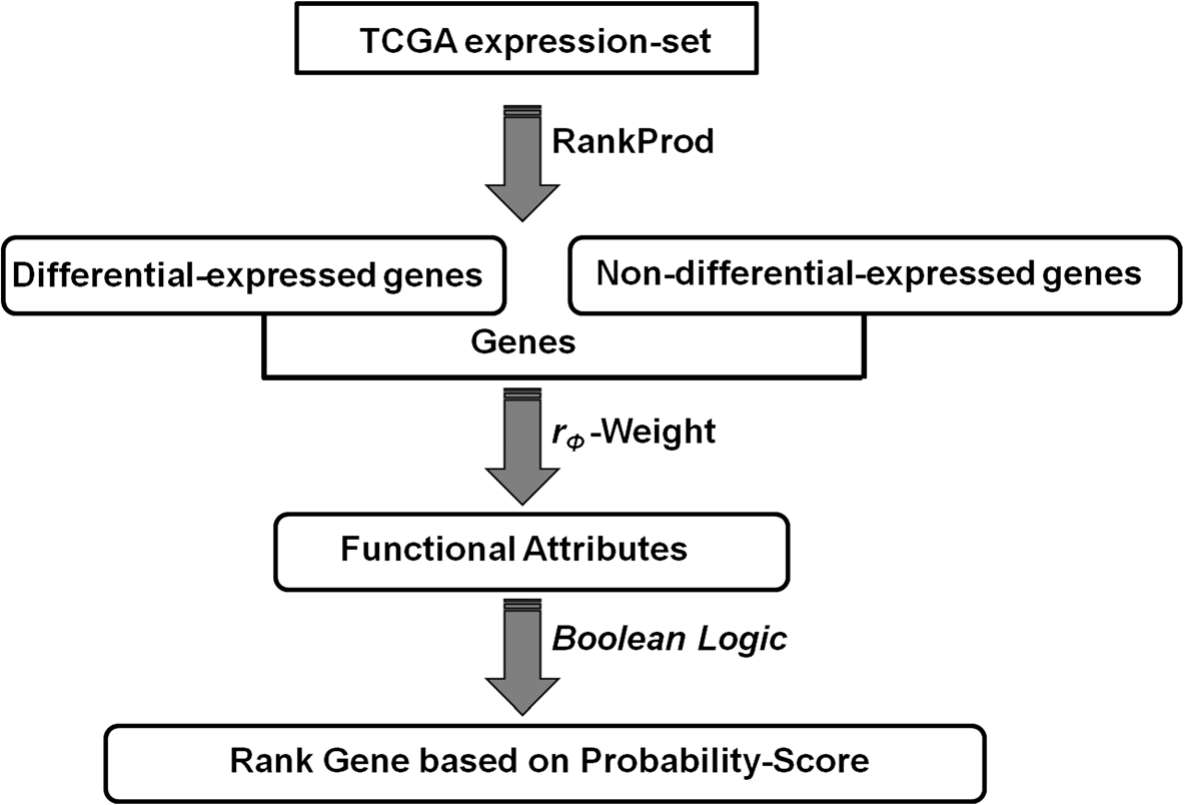
**Ranking genes in a Boolean logic framework.** Schematic representation of the workflow used to rank genes in a Boolean framework for identifying potential biomarkers in ovarian cancer.

In this study, an integrated systems approach is used to identify diseased-associated genes that are either not reported or poorly characterized in the ovarian tumor samples. We have estimated weights for the functional attributes associated with the known cancer gene list. These weights are then combined using a Boolean logic schema, to calculate the probability-based rank associated with differentially and non-differentially expressed genes. Finally, we have mapped high scoring ranks of differentially expressed genes on the co-expression gene interaction network to validate disease-associated genes (Figure 1). This study suggests that of the 17 shortlisted genes flagged as significant, the overexpressed genes *IRAK1*, *CHEK1* and *BUB1* may play an important role in ovarian cancer. Using survival analysis, we also report that the set of three genes, *CHEK1, AR,* and *LYN*, can be used in the prognosis of ovarian tumors.

## Methods

### Identification of differentially expressed genes

We extracted and analysed TCGA (The Cancer Genome Atlas) level 3 (Batch 9) ovarian serous cystadenocarcinoma data from the Affymetrix platform [17]. TCGA gene expression data are normalised, annotated and validated for expression variation relevant to the tissue types and with the type of array platforms, thus increasing the robustness in analysing expression data. Rather than a fold-change, we have calculated the differential expression of each gene by considering the percentage of false prediction (*pfp*) ≤5% using the RankProd R package [18]. RankProd uses the rank product non-parametric method to indentify up/down-regulated genes under one condition against the other (in our case tumor *vs.* normal ovarian samples). This is based on the null hypothesis that the order of all items is random and the probability of finding a specific item among the top *r* of *n* items in a list is *p* = *r/n*. Multiplying these probability leads to the identification of the rank product $\mathrm{RP}=\prod_{i}\frac{r_{i}}{n_{i}}$, where *r*_*i*_ is the rank of the item and *n*_*i*_ is the total number of items in the *i*^*th*^ list. The smaller the *RP* value, the smaller the probability that the observed placement of the item at the top of the list is due to chance.

### Relevant functional attributes in the disease condition

Although microarrays measure the relative abundance of mRNA transcripts, their translated proteins are likely to be differentially present in diseased tissue. Moreover, the extent of differential protein concentration under the disease condition is quite difficult to estimate due to the heterogeneity of cells in the tumor sample. Therefore, we considered a Boolean combination of six proteins functional attributes for searching genes associated with ovarian cancer, where the causative effects are not additive but combinatorial as well as non-linear. These functional attributes are tissue specificity (TS), transcription factors (TFs), post-translation modifications (PTMs), protein kinases (PKs), secreted proteins (SPs) and whether the protein is a hub in the interactome, with node connectivity greater than four (i.e. node connectivity > = 5) along with the gene attribute of methylation (METH), in cancer *vs.* non-cancer associated genes.

We hope to capture the underlying enabling factors for cancer, by considering the above protein functional attributes. Large-scale data analysis supports the fact that disease genes are generally tissue-specific and are over-expressed in those tissues where changes in gene expression result in pathology [19]. TFs are DNA-binding proteins regulating gene expression and thereby control cell development, differentiation and growth [20] and their aberrant activity has been implicated in the cancer disease condition [21]. Oncogenic conversion of normal cells into cancerous cells involves changes in transcription factor, e.g. c-Fos component of TF c-Jun/JUN/AP-1 is crucial for the estrogen receptor α (ERα) mediated transcription in breast cancer [22]. PTMs of key regulatory or structural proteins are known to play an important role in the progression of cancer by activation of signalling pathways, enhanced proliferation and impaired cell division and death [23]. PTMs contributing to tumorigenesis include phosphorylation, acetylation, methylation, glycosylation, prolyl isomerisation, hydroxylation, oxidation, glutathionylation, sumolyation and ubiquitination. For example, clinical evidence suggests that phosphorylation, acetylation and sumolyation of ERα lead to prostate and breast cancer in humans [24]. PKs are important signalling molecules for maintaining normal tissue architecture and function, hence mutation in these genes are a common cause of human cancer (http://www.sanger.ac.uk/genetics/CGP/Kinases/) [25]. Recent developments in proteomic analyses suggest an increasingly large number of genes overexpressed in ovarian cancer, of which several encode secreted proteins [26]. For example, the high expression of prostasin [27] and osteopontin [28] are recorded in the serum of ovarian cancer patients. Highly connected proteins, i.e. hubs are shown to be essential in connecting diverse functional modules inside the cell [29]. Also, epigenetic inactivation of tumor-suppressor genes due to methylation is well known in carcinogenesis [30].

### Data integration from multiple experiments

We extracted functional attributes *via* a text-mining approach. The cancer gene list was obtained by combining data from the Atlas of Genetics and Cytogenetics in Oncology and Haematology [31] and Futreal *et al.*[32], while information related to secreted proteins, tissue-specificity and protein’s post-translation modifications was obtained from HPRD [33]. Human protein kinases were extracted from the Human Kinome [34]. Transcription factors were extracted from TRED [35], HPRD [33] and TargetMine [36] databases. Gene methylations in ovarian samples were extracted from the studies reported by Mankoo *et al.*[37]. We considered the presence/absence of interaction in our high-confidence (HC) interactome dataset (detailed below) for differentially expressed genes, as biological pathways and networks of protein interactions are key paradigms to link molecules to biological functions. Therefore, interaction data were collected from BIND [38], BioGrid [39], DIP [40], HPRD [33], IntAct [41] and MINT [42] databases and merged into a single coherent interaction set after removing duplicate entries. Human protein interaction networks were further analysed to create a HC dataset by considering true interaction protein pairs as follow:

1. If binary interaction among proteins is known to be present in more than one databases.

2. Interacting protein pairs are true, if the interaction is verified from more than one detection method such as biochemical, biophysical, imaging techniques and/or protein complementation assay (PCA).

3. If interacting protein pairs have known protein domain interaction mentioned in 3did [43] and iPfam [44] databases.

4. PMIDs [45] were used as a proxy to support true interactions confirmed by more than one independent study.

These filters were used to define a HC protein interaction set to study the network properties of molecular functions and biological processes of interacting proteins. In this study, scoring schema for interactions were considered for those protein nodes with more than four interactions, as this is the empirical value of hubs suggested in gene co-expression stability in the analysis of protein interaction networks [46]. Therefore, we weighted such highly connected protein nodes encoded by the known cancerous genes.

### Weighting schema for Boolean-based probability calculation

We used phi-correlation (*r*_*Φ*_) as a measure of association between the functional attributes of the cancerous genes. This is one of the powerful methods to detect the association strength between two categorical data having binary values. Moreover, computationally it is related to the chi-square (*χ*^*2*^) value:

(1) $$r_{\varphi }=\sqrt{\frac{\chi^{2}}{N}}\text{,}$$

where *N* is the total number of genes.

### Scoring schema on the weighted functional attributes for ranking genes

We used the Boolean algorithm proposed by Nagaraj and Reverter [15] for ranking the differentially expressed genes in ovarian samples, with our own set of Boolean variables representing relevant functional attributes in the disease condition. The particular combination across the seven Boolean variables i.e. functional attributes for a given differentially and non-differentially expressed genes, was decomposed into its root. For example, if a given gene has four known functional attributes, then 2^4^ Boolean states are known to exist containing (2^4^-1) roots, i.e. all possible combinations of Boolean states at the positions of known functional attributes, excluding the Boolean value with all zero status. The probability of each root is simply the average sum of all the weights associated with known functional attributes calculated *via r*_*Φ*_. These root probabilities are then used to rank the differentially and non-differentially expressed genes by summing up all the probability values associated with the individual roots.

### Validation set

We retrieved the raw expression data for 153 ovarian tumor samples from the Gene Expression Omnibus entry GSE1349, containing samples in four tumor stages [9]. Raw expression values for each probe were transformed to log-scale with base 2. Probe IDs were converted to Entrez Gene IDs using AILUN [47]. For genes with multiple probes, the probes with the highest variance across the samples were used to describe the expression value for the genes. Probes with multiple or without Gene IDs were removed from the analysis. Pearson’s correlation coefficients were calculated based on the co-expression values alone, to define the pairwise gene co-expressions. We have taken a Pearson’s coefficient > 0.5 to define a link between co-expressed genes in the gene expression network.

### Network analysis of human signalling

We performed network analysis using the manually curated human signalling network [48]. The signalling network was pruned to contain associations between proteins alone and hence, small molecules were removed from the network, resulting in 1522 protein nodes and 4276 edges. The R package, igraph [49] was used for the network analysis. The Ingenuity Pathway Analysis system, (IPA, http://www.ingenuity.com) was further considered, to interpret the interaction of cancerous genes in humans.

### Clinical characterization using survival analysis

For the high scoring Boolean-based differentially expressed genes, we performed Kaplan-Meier survival analysis using the Cox-Mantel log-rank test, implemented as an R package. The significance of these genes in the normal and tumor samples were evaluated from the Welch two sample *t*-test. A less conservative *P-*value < 0.1 was considered for the statistical significance of genes in the ovarian tumor sample classification. The clinical data were downloaded from the TCGA data portal.

## Results and discussion

**Figure 2:**
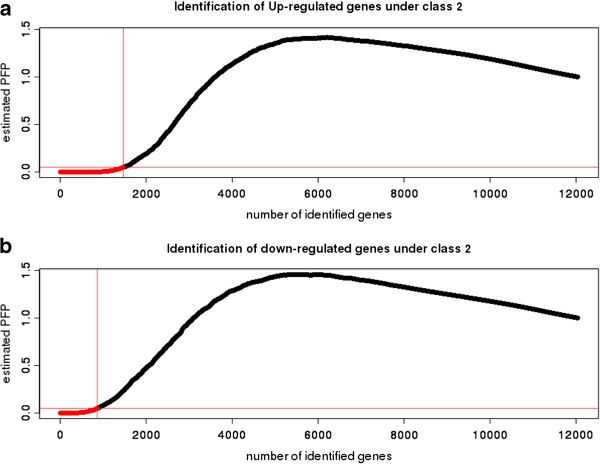
**Differential gene expression in the TCGA ovarian dataset.** Affymetrix TCGA gene expression dataset in ovarian tumor samples (class 1) *vs.* normal samples (class 2): **A**. Up-regulated genes and **B**. Down-regulated genes. RankProd analysis of differential gene expression at a percentage of false prediction (*pfp*) ≤ 5% is shown.

We used a systems biology approach to integrate diverse data resources as described in the Methods section. 2157 genes were identified to be differentially expressed in the tumor condition using the RankProd R package at a percentage of false positives: *pfp* ≤ 5%. The rank product method ensures ranking of expressed genes within each replicate (i.e. individual sample) and then computes the product of ranks across the replicates (i.e. multiple samples). Its distribution is then estimated by randomly permuting the observed ranks. Using this distribution, *pfp* is estimated. A cutoff of *pfp* ≤ 5% ensures that the observed data falls within two standard deviations of the mean, effectively translating to a *p-value* ≤ 0.05, expressing the probability that results at least as extreme as the above thresholds obtained in a sample were not due to chance. A total of 11,173 genes were considered in the TCGA expression set. This analysis suggested that 1353 and 804 genes were up-regulated and down-regulated respectively (Figure 2 and Additional file 1). An estimation of the weight was carried out *via* a simple observation of known functional attributes present between cancerous and non-cancerous genes. Table 1 lists the different functional attributes used as weights in this study. An odds-ratio analysis of differentially and non-differentially expressed genes showed no apparent differences (Additional file 2). This suggests that no single functional attribute can be selected alone in the classification of genes as a potential biomarker for the prognosis of the ovarian tumor condition. Moreover, cancer is well established as a disease model where the cellular system is abnormal leading to an uncontrolled cell division. Hence, a synergistic approach is needed to encapsulate the various functional attributes together for the understanding of the cancerous state. Figure 1 illustrates the workflow used for ranking genes. A Boolean framework for measuring unknown interactions between different biological entities and for the classification of genes in disease conditions have been reported by earlier studies [12,15].

**Table 1:** **Phi-correlation (*r***_***Φ***_**) weights calculated for the functional attributes such as methylation, post-translation modifications, protein kinase, secretory proteins, tissue-specificity, protein interaction nodes with connectivity > =5 and transcription factor in cancerous *vs. *non-cancerous genes associated with ovarian cancerous tumor samples**

**Functional Attributes**	**Phi-correlation value**	***P-value***
Methylation	0.021944	0.0803
Post-translation modifications	0.046598	0.0004
Protein kinase	0.037870	0.0030
Secretory proteins	0.036727	0.0026
Tissue specificity	0.038675	0.0019
Interactome (node connectivity > =5)	0.072986	0.0001
Transcription factor	0.048745	0.0002

**Figure 3:**
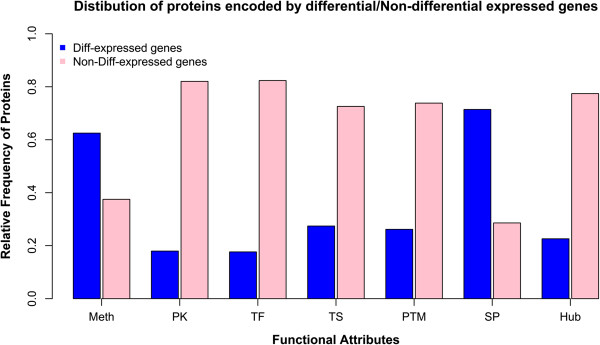
**Functional attributes presented in various proteins encoded by differential/non-differential gene expression in the TCGA data.** Histogram representing functional attributes such as Meth (methylation), PK (protein kinase), TF (transcription factor), TS (tissue specificity), PTM (post-translation modification), SP (secreted-proteins) and Hub (protein interactions where node connectivity > =5) presented in proteins encoded by differentially (in blue)/non-differentially (in pink) expressed genes.

In this study, seven functional attributes, such as epigenetic inactivation (CpG gene methylation), protein post-translation modification, protein kinase, secreted protein, tissue-specificity, transcription factor and hub proteins in an interactome (protein node connectivity of 5 and above) were considered for the classification in the Boolean logic framework. We defined the Boolean logic for each gene, corresponding to the selected functional attributes (Table 2 and Additional file 2). These Boolean values were then decomposed to their roots to calculate the overall probability based on their functional attribute weights (detailed in the Methods section). Nagaraj and Reverter [15] have reported an average Boolean probability score of 0.219 (ranging from 0.002 to 0.687) for known cancer genes, compared to an average score of 0.081 (ranging from 0.000 to 0.589) for the other genes, indicating an average 2.71-fold enrichment using their Boolean logic, in their exhaustive study of 21,892 genes in colorectal cancer. In order to identify differential and non-differential gene expression as potential biomarkers with high confidence, we have set an empirical probability score greater than 0.5 as a cut-off, which is more than twice their reported average Boolean probability score. At this cut-off value, we were able to identify 17 differentially expressed genes (Table 2), whereas non-differential expression is noted for 48 genes (Additional file 3). In the TCGA expression dataset, we found seven (*IRAK1*, *STC2*, *CDC7*, *CHEK1*, *KLK6*, *BUB1* and *CHEK2*) and ten (*IGF1R*, *DAB2*, *IGFBP7*, *FOXL2*, *LCN2*, *CLU*, *LYN*, *PGR*, *AR* and *VIM*) genes to be up-regulated and down-regulated, respectively, using RankProd analysis. In the validation dataset genes *IGFBP7* and *LCN2* are absent. Figure 3 compares the known functional attributes present in proteins encoded by differentially and non-differentially expressed genes. Moreover, we have verified the importance of these differentially expressed genes by mapping to their biological pathways (Additional file 4).

**Table 2:** Boolean-based probability scores for ranking the 17 differentially expressed genes

***Gene symbol***	***Gene ID***	***Up***	***Down***	***Boolean values***	***Rank***
*KLK6*	5653	1	0	1011001	0.697808
*IRAK1*	3654	1	0	0111010	0.607561
*CDC7*	8317	1	0	0111010	0.607561
*CHEK1*	1111	1	0	0111010	0.607561
*BUB1*	699	1	0	0111010	0.607561
*CHEK2*	11200	1	0	0111010	0.607561
*STC2*	8614	1	0	1011010	0.584684
*DAB2*	1601	0	1	0011011	0.743532
*VIM*	7431	0	1	0011011	0.743532
*FOXL2*	668	0	1	0011101	0.735481
*LCN2*	3934	0	1	1011001	0.697808
*PGR*	5241	0	1	0011110	0.644578
*AR*	367	0	1	0011110	0.644578
*IGF1R*	3480	0	1	0111010	0.607561
*LYN*	4067	0	1	0111010	0.607561
*IGFBP7*	3490	0	1	1011010	0.584684
*CLU*	1191	0	1	1011010	0.584684

### Protein kinases

Protein kinases are important regulators of cell function and belong to a functionally diverse gene family. They affect the activity, localisation and overall function of other proteins by adding a phosphate group and thereby control the activity of cellular processes. Kinases are particularly important in signal transduction and co-ordination of complex functions such as cell cycle and pathological conditions. Identification of *IRAK1* as a differentially expressed gene in ovarian cancer suggests its important role in this disease. It is a putative Ser/Thr kinase known to partially interact with transcription factor, NF-κB. Activation of NF-κB leads to cell proliferation, survival and migration [50]. Over-expression of this gene suggests indirect cell survival and proliferation in the ovarian tumor condition. Similarly, *IGF1R* is a receptor with tyrosine kinase activity, which binds an insulin-like growth factor. It is over-expressed in most malignant tissue, acting as an anti-apoptotic agent by enhancing cell survival [51,52]. *LYN* is a non-receptor tyrosine kinase, phosphorylating caspase 8, rendering it inactive and thereby assisting apoptosis of the inflammatory cell [53]. In the absence of the normal expression of *LYN,* active caspase 8 may prevent the tumor cells from undergoing apoptosis.

**Figure 4:**
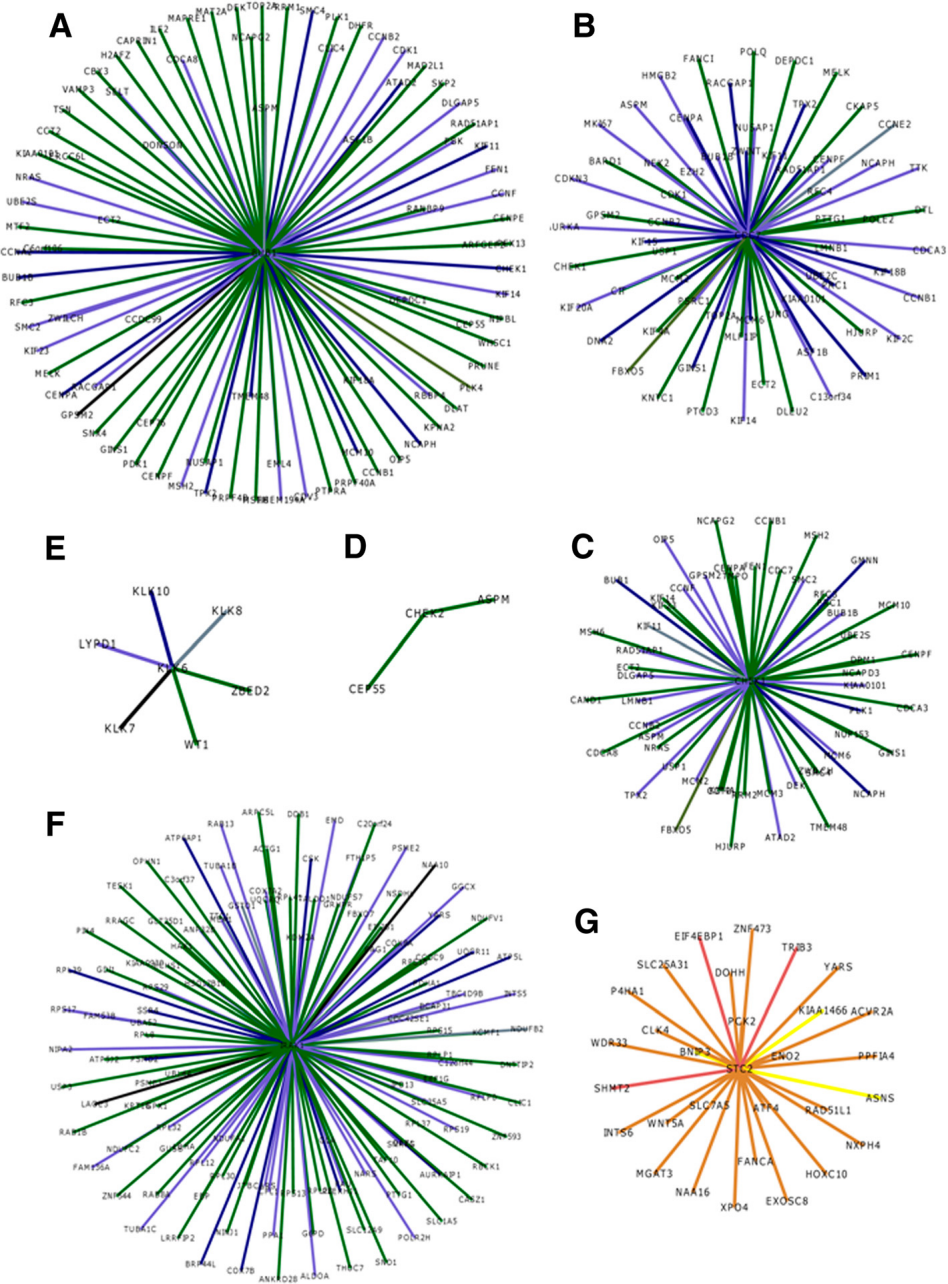
**Co-expression of up-regulated genes.** Schematic representation of up-regulated. Schematic representation genes: **A**. *BUB1*, **B**. *CDC7*, **C**. *CHEK1*, **D**. *CHEK2*, **E**. *KLK6*, **F**. *IRAK1* and **G**. *STC2*. Edges are colour-coded to highlight the range of pearson’s correlation coefficient in co-expression network: black (> 0.7), slate grey (0.65 - 0.7), navy blue (0.60 - 0.65), slate blue (0.55- 0.60), dark green (0.50 – 0.55), dark olive green (0.45 - 0.05), yellow (0.40 – 0.45), indian red (0.35 -0.40), peru (0.30 -0.35). Refer to Additional file 5 for co-expressed neighbors and their associated co-expression Pearson’s correlation values.

Other important kinases in cell survival and proliferation during tumorigenesis are associated with key cell cycle proteins. *CDC7* (cell-division cycle 7 homolog of *S. cerevisiae*) and *BUB1* (budding uninhibited by benzimidazoles 1 homolog of *S. cerevisiae*) encode protein kinases which induce G1/S transition and are involved with the spindle checkpoint function, respectively during cell mitosis. *CDC7* is known to be overexpressed in the epithelial ovarian carcinoma, resulting in tumor progression, genomic instability and accelerated cell division [54]. On the other hand, *BUB1* overexpression induces aneuploidy and tumor formation [55]. *CHEK1* (checkpoint kinase 1) is an another important cell-cycle molecule of Ser/Thr protein kinase family mediating signals from ATM and ATR cell cycle proteins involved in the DNA damage response and associated with chromatin in the meiotic prophase I. The importance of this protein in tumor invasiveness has been suggested by researchers in lung, bladder, liver, prostate, gastric, brain, cervical and colorectal cancers and B-cell lymphoma [56-58]. *CHEK2* (checkpoint kinase 2) is yet another important cell cycle protein which regulated key proteins during cell division. It interacted with *BRCA1* (breast cancer 1) to restore survival in response to DNA damage with known association with endometrial cancer risk [59]. We observed overexpression of *IRAK1, BUB1*, *CDC7*, *CHEK1* and *CHEK2* genes in TCGA samples at a high Boolean probability score of 0.607561, together with the co-expression of other key cell-cycle molecules in an independent validation expression set GSE1349 suggesting their association in ovarian cancer (Figure 4 and Additional file 5). The presence of high probability up-regulated genes in the co-expression network (Pearson’s correlation coefficient > 0.5) is shown in Additional file 6. The co-expression network of downregulated genes is available from Additional file 7.

### Serine proteases

Serine proteases are proteolytic enzymes, hydrolysing the peptide bond of protein substrates *via* a nucleophilic serine residue in the active site [60]. Serine proteases play diverse roles in human health, from non-specific digestion to highly regulated functions like embryonic development, immune response and blood coagulation. Moreover, insufficient or excess protease activity can promote significant pathologies like cancer, inflammation, hemophilia, heart attack, stroke, pancreatitis and parasite infection [61]. We suggest the potential use of *KLK6* (kallikrein-related peptidase 6) as a potential biomarker for ovarian cancer based on its high Boolean probability score (0.697808) (Figure 4). *KLK6* is a serine protease with diverse functional roles inside the cell. It has been suggested that overexpression of this protein leads to the loss of cell-cell adhesion in skin cancer (melanoma) [62]. Moreover, a recent study reports the up-regulation of *KLK6* in colon cancer and its use as a potential biomarker and therapeutic agent [63].

### Secreted proteins

Secreted proteins are secreted from the cell into the extracellular space and have important biological regulatory roles with the potential for therapeutics. *STC2* (Stanniocalcin 2) is a secreted homodimeric glycoprotein that is expressed in a variety of tissues. *STC2* is known to promote the epithelial-mesenchymal transition and invasiveness in human ovarian cancer under inadequate oxygen supply to the tissue [64]. Our results show that STC2 is a significant up-regulated gene, promoting ovarian cancer. On the other hand, *CLU* (clusterin) and *LCN2* (lipocalin 2) are down-regulated genes in our analysis. *CLU* encodes a protein which is secreted under stress conditions, that functions as a strong anti-migratory and anti-invasive agent by inducing the destruction of the actin cytoskeleton inside the cell [65]. The decreased expression of *CLU* thus promotes the cancerous disease condition. *LCN2* encodes a 25 kDa secretory protein involved with iron-transportation and contributes to endometrial carcinoma [66]. Moreover, it is a key molecule in various signalling pathways (Additional file 4). Down-regulation of *LCN2* due to epigenetic inactivation may lead to ovarian carcinoma.

### Other types of proteins

We observed down-regulation of genes with high probability associated with phosphoproteins, transcription factors and receptors due to epigenetic inactivation. Phosphoprotein *DAB2* is a mitogen-responsive agent, acting as tumor suppressor in normal ovaian epithelial cells and down-regulation of this gene modulates the TGF-β signalling pathway [67]. *FOXL2* (forkhead box L2) encodes a transcription factor which helps in the normal development of ovarian tissue. *IGFBP7* (insulin-like growth factor binding domain) is known as the tumor suppressor gene, leading to lung cancer due to the epigenetic inactivation [68]. *PGR* (progesterone receptor) encodes a protein playing a central role in the reproductive system by maintaining progesterone levels and ensuring normal pregnancy. *AR* (androgen receptor) encodes a protein which functions as a steroid hormone-activated transcription factor and has been shown to be involved in prostate cancer [69] as well as in ovarian cancer in association with *p44*[70]. *VIM* (vimentin) encodes a protein that is responsible for maintaining cell shape, integrity of the cytoplasm and stabilizing cytoskeleton interaction. Thus, the decreased expression of these genes could be indicative of ovarian cancer.

### Relevance to cancer

We have mapped these 17 differentially expressed genes to gene ontology (GO) biological process terms collated from the GATHER [71] and the GENECARDS [72] databases as well as from the recent literature. The relevant GO terms linking these genes to the cancer hallmarks described by Hanahan and Weinberg [6] are presented in Table 3, with detailed information in Additional file 8 and Additional file 9. Each hallmark is associated with 1-13 of the 17 differentially expressed genes (mean = 5.7) while each gene maps to 1-6 hallmarks (mean = 2.8). While almost all the GO biological process terms could be unambiguously mapped to a cancer hallmark, the regulation of apoptotic process (GO:0042981) for *LYN* maps to both hallmark 3: active invasion and metastasis and hallmark 6: resist cell death and is shown in italics in Table 3. For *STC2* and *LCN2,* the GeneCards biological process GO terms were augmented with literature search and the relevant references are provided in Additional file 9.

**Table 3:** GO biological process terms of 17 differentially expressed genes mapped to the hallmarks of cancer

**Gene/cancer hallmark**	**HM1**	**HM2**	**HM3**	**HM4**	**HM5**	**HM6**	**HM7**	**HM8**	**HM9**	**HM10**
*KLK6*		GC			G, GC					G, GC
*IRAK1*		GC		GC				GC		GC
*CDC7*	G	G,GC		G, GC			GC	G		
*CHEK1*	G	G		G			G, GC	G		
*BUB1*				G			GC			
*CHEK2*				G, GC			G, GC			
*STC2*	GC	GC	L	L						
*DAB2*	GC			G, GC	GC			GC		
*VIM*			GC			GC				
*FOXL2*				GC	GC	GC	GC			
*LCN2*			L			L				
*PGR*	GC									
*AR*	GC	GC		G, GC						
*IGF1R*		G, GC	G, GC	G, GC		G, GC		G	GC	
*LYN*	GC		*GC*	GC		*GC*	GC		GC	
*IGFBP7*	G, GC	G		G, GC				G		
*CLU*			GC	GC					GC	

HM1: sustain growth signal, HM2: escape growth suppressor i.e. insensitivity to growth inhibitor, HM3: active invasion and metastasis, HM4: limitless replicative potential/enable replicative immortality, HM5: induce angiogenesis, HM6: resist cell death, HM7: cause genomic instability, HM8: deregulate cellular energetic, HM9: avoid immune destruction, HM10: tumor promoting inflammation, G: GATHER [71], GC: GeneCards [72], L: literature (G details in Additional file 8 and GC and L details in Additional file 9).

**Figure 5:**
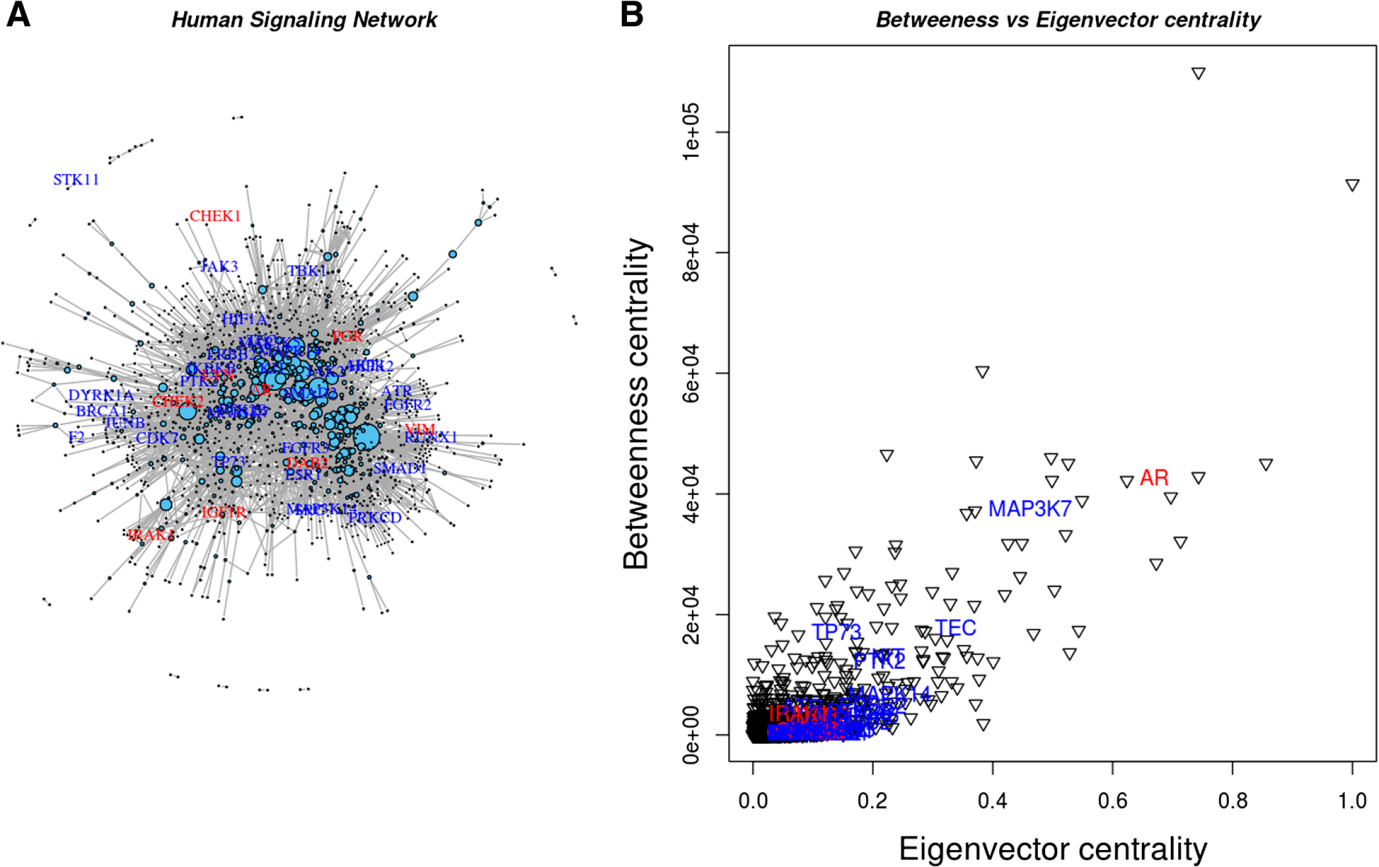
**Differentially and non-differentially expressed genes with high Boolean scores mapped to the human signalling network. A.** Mapping of differentially expressed (with red labels) and non-differentially expressed (with blue labels) expressed genes from the TCGA data set on the human signalling network. Node size represents the residual value of linearly regressed betweenness and eigenvector centralities. **B.** Betweenness *vs*. eigenvector centrality plot of nine differentially expressed and 32 and non-differentially expressed genes identified in the human signalling network.

**Figure 6:**
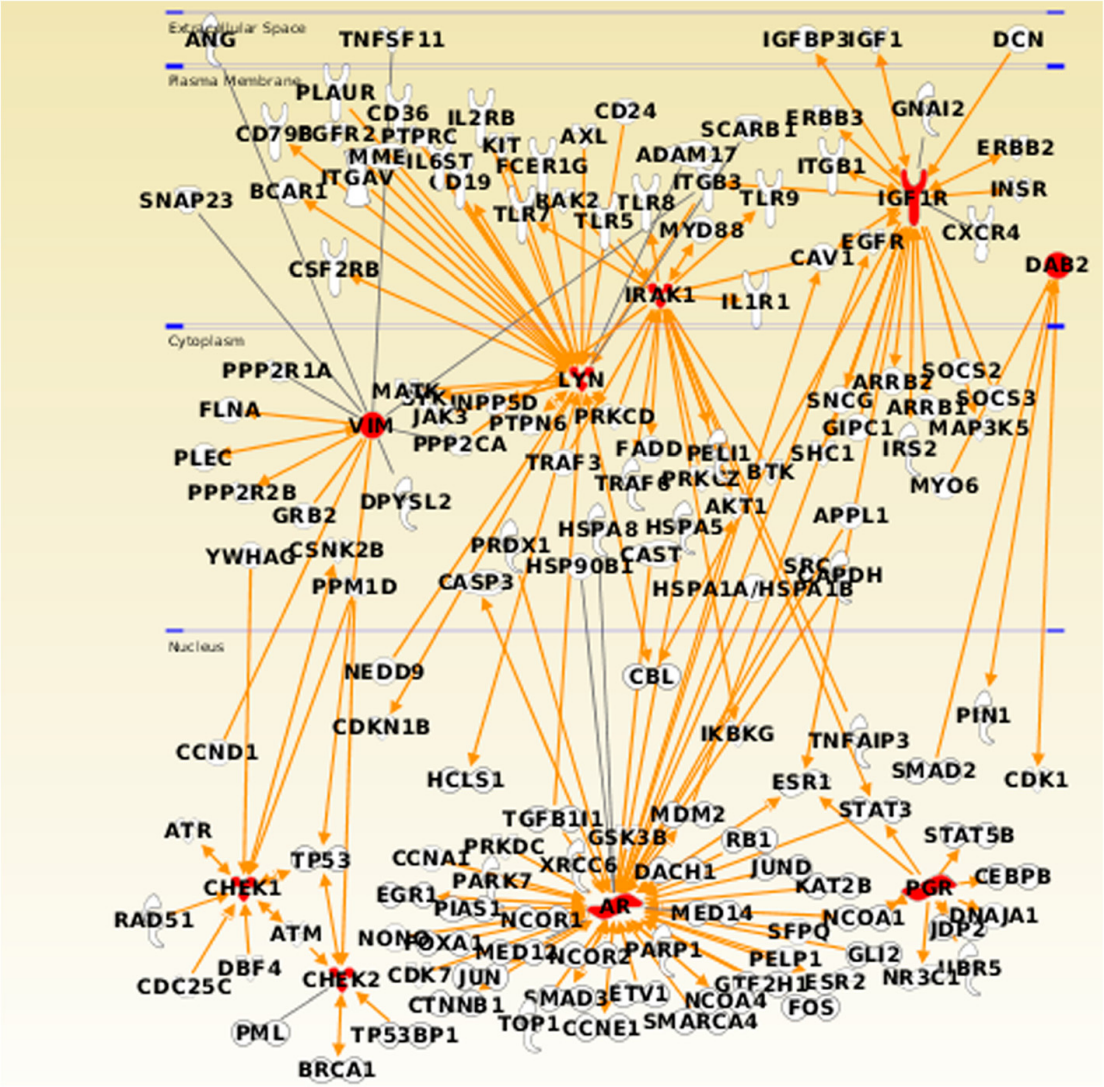
**Ingenuity Pathway Analysis visualization of oncogene interactions.** Interaction of nine differentially expressed genes with high Boolean scores in the human signalling network mapped to known oncogenes in the Ingenuity Pathway Analysis knowledge-based expert system.

The complexity of cellular regulation is encapsulated in the signalling network. Moreover, signalling network also helps to understand the dynamics of oncogenic cross-talk by determining the sites at which oncogenic signals occur and through which oncogenic signals are transduced. Therefore, differentially and non-differentially expressed genes with high Boolean scores identified in the TCGA dataset (listed in Table 2 and Additional file 3, respectively) were mapped to the manually curated human signalling network [48], shown in Figure 5. Nine of the 17 differentially expressed genes and 32 of 48 non-differentially expressed genes could be mapped to the signalling network (Figure 5A). We have computed the importance of a node in the signalling network using betweenness centrality (the number of times a node acts as a bridge along the shortest path between two other nodes) and the influence of a node in the network using eigenvector centrality (relative scores to all nodes in the network with connections to high-scoring nodes receiving higher scored compared to low-ranking nodes). A plot of the two signalling network centrality measures (betweenness centrality and eigenvector centrality; Figure 5B) suggests the importance of the differentially expressed *AR* and the non-differentially expressed *MAP3K7* (mitogen-activated protein kinase kinase kinase 7, involved in stress response) genes, which have ten shared interactions, from BioGraph [73], ranked 636 out of 18180 ‘Gene’ concepts (top 3.50%) in BioGraph’s knowledge base. Moreover, the nine differentially expressed genes (*AR*, *CHEK1*, *CHEK2*, *PGR*, *VIM*, *LYN*, *IRAK1*, *IGF1R* and *DAB2*) mapped to the signalling network were further identified using the Ingenuity Pathway Analysis system to visualize the interaction of these genes with the known oncogenes (Figure 6). The central role played by *CHEK1* in the DNA damage response signalling network, has been confirmed by Dai and Grant [74], where *CHEK2, CDC7* and *BUB1* have also been identified from the 17 differentially expressed genes reported here.

### Clinical characterization

**Figure 7:**
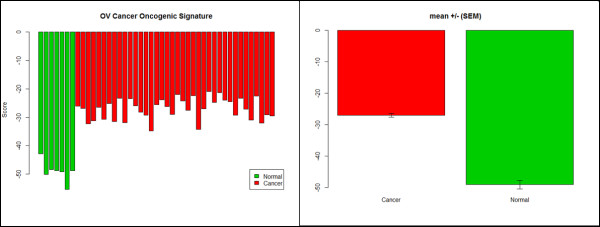
**Ovarian cancer gene signature.** Gene signature constructed from the expression values for each of genes given in Table 2, by substracting the sum of the down-regulated gene expression values from the sum of the up-regulated gene expression values. **A**. Individual scores for each of the normal and cancer patients. **B**. Mean signature values +/- (SEM) for the normal and cancer patients. Welch two sample *t*-test (t = -14.69, df = 8.45, p=2.621E-07).

Table 2 lists 17 genes, of which seven are up-regulated and ten are down-regulated in ovarian cancer patients. The expression patterns of these genes suggest that the sum of the up-regulated gene expression values minus the sum of the down-regulated gene expression values should be maximized in ovarian cancer patients compared to controls without ovarian cancer (Additional file 10). Figure 7 shows that this is indeed the case for the 38 ovarian clinical samples and seven normal samples in this dataset and that this simple formula for the 17 genes identified here can be used to successfully distinguish between normal and ovarian cancer patients (*p-value* < 1.2E-06).

**Figure 8:**
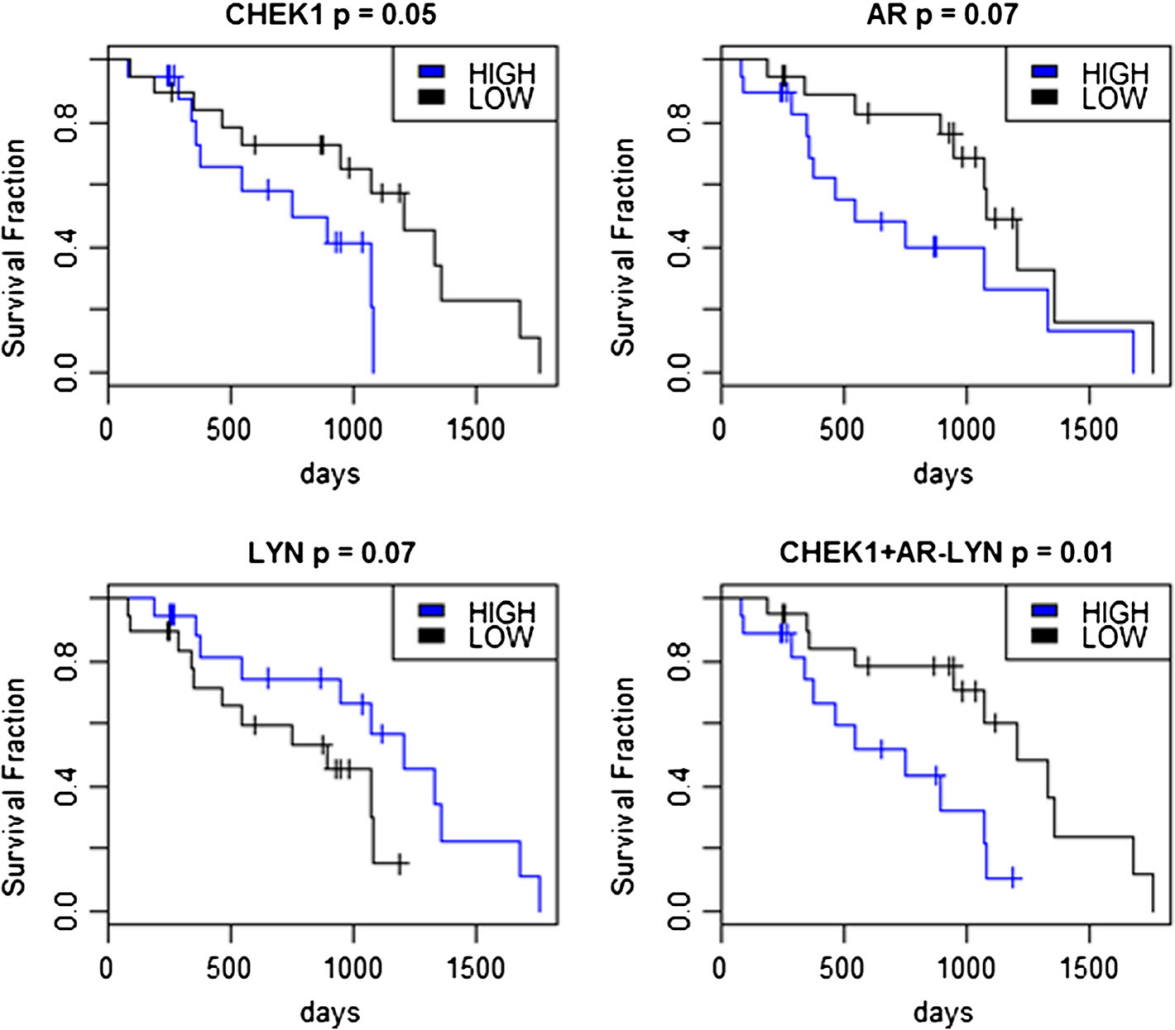
**Survival curves for ovarian cancer patients designated as being either high or low expression patients for genes *CHEK1, AR *and *LYN*.** The lower of the two lines in each survival plot indicates patient with poor prognosis. The combinational plot CHEK1 + AR-LYN represents the sum of the expression values of *CHEK1* and *AR* minus the expression of *LYN*. The p associated with each plot gives the *p-value* from the log-rank test for equality between the low and high expression groups from R’s Kaplan-Meier estimate of survival.

Survival analysis was carried out to suggest if any of above 17 genes or their combinations, can be used in the classification and prognosis of ovarian cancer, to classify good and poor prognostic tumors. To demonstrate the survival analysis across the 38 ovarian clinical samples in this dataset, expression levels of each of the 17 genes were ranked from lowest to highest expression. Tumor samples associated with the lower 50% of the expression values for a given gene were labelled as “low-expression” for that gene; otherwise, they were labelled as a “high-expression” sample for that gene. Log-rank tests were then performed to suggest the difference between expected *vs.* observed survival outcomes for the low- and high-expression tumor samples for each of the genes. As there were only 38 ovarian tumor samples with clinical data, we chose the less stringent log-rank *P-value* of 0.1 and discovered three genes, *CHEK1, AR* and *LYN* exhibit a prognostic value, based on this cut-off level (see Figure 8).

In Figure 8, the lower of the two curves in each of the four survival analysis plots indicates tumor samples associated with poor prognosis. Interestingly, though the survival curves associated with gene *AR* indicate poor prognosis is expected for tumor samples within the high expression range of *AR*, from Table 2 we note that *AR* is down-regulated in ovarian cancer. From Figure 8, it is seen that high expression for up-regulated *CHEK1* and down-regulated *AR* and low expression for *LYN* leads to poor prognosis. The clinical data thus suggests a preference for limited down-regulation of *AR*. Therefore, combining the expression levels of these three genes as *CHEK1+AR-LYN* (Figure 8), then ranking this score from lowest to highest values and associating the patients into low and high expression groups, as before, gave greater significance in the prognostic outcome for classifying good and poor tumour outcomes than did the individual genes. Biologically, this combination represents increased cell cycle control, particularly for entry into mitosis (*CHEK1),* decreased expression of the androgen receptor (AR), whose expression levels have controversial reports as a favourable prognostic factor in epithelial ovarian cancer [75,76] and moderately decreased expression of *LYN,* resulting in apoptosis of tumor cells.

## Conclusions

We have statistically integrated gene expression and protein interaction data by combining weights in a Boolean framework to identify high scoring differentially expressed genes in ovarian tumor samples. This has resulted in the identification of important genes associated with critical biological processes. We identified 17 differentially expressed genes from a dataset of 11,173 genes, where seven and ten genes were up- and down-regulated, respectively with significant probability score in a Boolean logic schema. We report three genes (*IRAK1*, *CHEK1* and *BUB1*) to be significant in ovarian tumor samples for the first time, to the best of our knowledge. A recent study on ovarian cancer supports our observation that the cell cycle proteins, *CHEK1* and *BUB1,* are over-expressed and are important to the tumor condition, lending support to our observation [75]. Our results demonstrate the significance of multiple data types and knowledge-guided integration of diverse biological information to understand the molecular mechanisms associated in ovarian cancer and their application in the discovery of biomarkers. Network analysis of the human signalling pathways suggests the importance of the *AR* gene, which is down-regulated in ovarian tumor samples, leading to cancer. We also showed that the expression levels of the 17 genes discovered in this analysis can be used to distinguish between normal and ovarian cancer patients and that three genes, *CHEK1, AR* and *LYN* in combination can be used to classify good and poor prognostic tumors [77] from ovarian cancer patients.

## Competing interests

The authors declare that they have no competing interests.

## Authors’ contributions

Conceived and designed the experiment: GK. Data collected and analysed: GK, SR. EB performed the clinical classification. Manuscript has been written and finalised by GK, EB and SR. All authors read and approved the final manuscript.

## Supplementary Material

Additional file 1 List of up- and down-regulated genes in the TCGA dataset. Click here for file

Additional file 2 Differential/Non-differential gene expression for various functional attributes. Click here for file

Additional file 3 Boolean-based probability score for ranking 48 non-differentially expressed genes. Click here for file

Additional file 4 **Statistically significant pathway analysis from the NCI-nature*PID*(Pathway Interaction Database) of the 17 differentially expressed genes in various biological pathways.** Click here for file

Additional file 5 High confidence up/down-regulated genes identified in the Boolean framework with their co-expressed neighbors. Click here for file

Additional file 6 **Schematic representation of co-expressed genes with significant Boolean values.** Edges are colour-coded to highlight the range of pearson’s correlation coefficient in co-expression network: black (> 0.7), slate grey (0.65 - 0.7), navy blue (0.60 - 0.65), slate blue ( 0.55- 0.60), dark green (0.50 – 0.55), dark olive green (0.45 – 0.50 ). Yellow (0.40 -0.45), indian red (0.35 -0.40 ) and peru (0.30 - 0.35). Click here for file

Additional file 7 **GATHER [**[66]**] GO biological process annotations of the 17 differentially expressed genes associated with the cancer hallmarks in Table**3**.** Click here for file

Additional file 8 **Relevant GO biological process characterization from GeneCards [**[67]**] for the 17 differentially expressed genes, mapped to cancer hallmarks (HM) in Table** 3**.** Click here for file

Additional file 9 Gene expression data for the 17 genes identified in this study across 45 (38 tumor + 7 normal) samples. Click here for file

Additional file 10 Gene expression data for the 17 genes identified in this study across 45 (38 tumor + 7 normal) samples. Click here for file
