# Supplementary material for: Identification of ovarian cancer associated genes using an integrated approach in a Boolean framework
Source: BMC Syst Biol. 2013 Feb 6;7:12. doi: 10.1186/1752-0509-7-12 (PMC3605242; doi:10.1186/1752-0509-7-12)
Supplement: Additional file 2 — Differential/Non-differential gene expression for various functional attributes. [file 1752-0509-7-12-S2.pdf]

Identification of ovarian cancer associated genes using an integrated approach in a Boolean framework
by Gaurav Kumar, Edmond J. Breen and Shoba Ranganathan

Additional File 2

Differential/Non-differential gene expression data analysis for the seven functional attributes.

Functional Attributes		Differential gene expression				Non-differential gene expression			
		Average Rank	Min.	Max.	Odd-Ratio	Average Rank	Min.	Max.	Odd-Ratio
**Secretome**	+	0.093	0.000	0.698	2.111	0.051	0.000	0.585	1.246
	-	0.044	0.000	0.744		0.041	0.000	0.776	
**Protein kinase**	+	0.216	0.037	0.608	5.194	0.196	0.037	0.739	5.515
	-	0.042	0.000	0.744		0.036	0.000	0.776	
**Tissue specificity**	+	0.112	0.038	0.744	3.647	0.102	0.038	0.744	3.768
	-	0.031	0.000	0.374		0.027	0.000	0.766	
**Post-translation modification**	+	0.131	0.039	0.744	6.389	0.126	0.039	0.766	6.517
	-	0.021	0.000	0.372		0.019	0.000	0.393	
**Transcription Factor**	+	0.172	0.047	0.735	3.909	0.169	0.047	0.776	4.584
	-	0.044	0.000	0.744		0.037	0.000	0.744	
**Protein-interaction (nodes >4)**	+	0.157	0.049	0.744	5.744	0.146	0.049	0.776	6.597
	-	0.027	0.000	0.735		0.022	0.000	0.369	
**Methylation**	+	0.187	0.073	0.744	4.388	0.148	0.000	0.776	3.807
	-	0.043	0.000	0.645		0.039	0.000	0.645	
