# Supplementary material for: Identification of ovarian cancer associated genes using an integrated approach in a Boolean framework
Source: BMC Syst Biol. 2013 Feb 6;7:12. doi: 10.1186/1752-0509-7-12 (PMC3605242; doi:10.1186/1752-0509-7-12)
Supplement: Additional file 3 — Boolean-based probability score for ranking 48 non-differentially expressed genes. [file 1752-0509-7-12-S3.pdf]

Identification of ovarian cancer associated genes using an intgrated approach in a Boolean framework

by Gaurav Kumar, Edmond J. Breen and Shoba Ranganathan

Additional File 3

Boolean-based probability score for ranking the 48 non-differentially expressed genes.

Gene symbol	*Gene-ID*	*Boolean values*	*Rank*
ESR1	2099	0001111	0.776265
PRLR	5618	0011011	0.743532
WEE1	7465	0101011	0.739246
SMAD2	4087	0011110	0.644578
CDC5L	988	0011110	0.644578
HIF1A	3091	0011110	0.644578
BRCA1	672	0011110	0.644578
JUNB	3726	0011110	0.644578
RUNX1	861	0011110	0.644578
BACH1	571	0011110	0.644578
MAX	4149	0011110	0.644578
GTF2A1	2957	0011110	0.644578
SREBF1	6720	0011110	0.644578
TP73	7161	0011110	0.644578
CDK7	1022	0101110	0.640294
AKT1	207	0111010	0.607561
HIPK2	28996	0111010	0.607561
ERBB2	2064	0111010	0.607561
KIT	3815	0111010	0.607561
JAK3	3718	0111010	0.607561
TBK1	29110	0111010	0.607561
PAK4	10298	0111010	0.607561
MAP3K14	9020	0111010	0.607561
BRD4	23476	0111010	0.607561
TRIM28	10155	0111010	0.607561
LATS1	9113	0111010	0.607561
MAPK14	1432	0111010	0.607561
STK11	6794	0111010	0.607561

Gene symbol	***Gene-ID***	***Boolean values***	***Rank***
TEC	7006	0111010	0.607561
FGFR1	2260	0111010	0.607561
STK16	8576	0111010	0.607561
MAP3K5	4217	0111010	0.607561
MAP3K7	6885	0111010	0.607561
IKBKB	3551	0111010	0.607561
PTK2	5747	0111010	0.607561
PTK2B	2185	0111010	0.607561
FGFR3	2261	0111010	0.607561
JAK2	3717	0111010	0.607561
ATR	545	0111010	0.607561
FLT1	2321	0111010	0.607561
FGFR2	2261	0111010	0.607561
DYRK1A	1859	0111010	0.607561
PRKCD	5580	0111010	0.607561
ERBB4	2066	0111010	0.607561
SRC	6714	0111010	0.607561
SERPINA1	5265	1011010	0.584684
SMAD1	5265	1011010	0.584684
F2	2147	1011010	0.584684
