# Supplementary material for: Identification of ovarian cancer associated genes using an integrated approach in a Boolean framework
Source: BMC Syst Biol. 2013 Feb 6;7:12. doi: 10.1186/1752-0509-7-12 (PMC3605242; doi:10.1186/1752-0509-7-12)
Supplement: Additional file 4 — Statistically significant pathway analysis from the NCI-naturePID(Pathway Interaction Database) of the 17 differentially expressed genes in various biological pathways. [file 1752-0509-7-12-S4.pdf]

Identification of ovarian cancer associated genes using an integrated approach in a Boolean framework

by Gaurav Kumar, Edmond J. Breen and Shoba Ranganathan

Additional File 4

Statistically significant NCI-Nature Pathway Interaction Database analysis of the 17 differentially expressed genes. PID is a database with biomolecular interactions and cellular processes assembled into authoritative human signalling pathways.

Gene symbol*	***Gene ID***	***PID-Pathways and associated P-Values***
AR	367	Regulation of nuclear SMAD2/3 signaling (1.88e-04); Nongenotropic Androgen signaling (2.80e-04); FOXA1 transcription factor network (1.06e-03); Coregulation of Androgen receptor activity (3.84e-03); Notch-mediated HES/HEY network (1.33e-02); Regulation of Androgen receptor activity (1.62e-02); Regulation of nuclear beta catenin signaling and target gene transcription (3.70e-01)
BUB1	699	p73 transcription factor network (1.25e-03); Aurora B signaling (5.66e-02); PLK1 signaling events (7.34e-02)
CHEK1	1111	p53 pathway (1.56e-07); p73 transcription factor network (1.25e-03); Circadian rhythm pathway (1.16e-02); Fanconi anemia pathway (1.33e-02); ATR signaling pathway (5.89e-02)
CHEK2	11200	p53 pathway (1.56e-07); ATM pathway (5.03e-03); PLK3 signaling events (6.08e-02); FOXM1 transcription factor network (6.37e-02)
CLU	1191	Validated targets of C-MYC transcriptional repression (8.87e-04)
DAB2	1601	TGF-beta receptor signaling (1.78e-02)
IGF1R	3480	IGF1 pathway (2.80e-04); Plasma membrane estrogen receptor signaling (8.91e-04); Integrins in angiogenesis (1.19e-03); SHP2 signaling (2.12e-02); Posttranslational regulation of adherens junction stability and disassembly (7.84e-02); Stabilization and expansion of the E-cadherin adherens junction (2.98e-01)
IRAK1	3654	IL1-mediated signaling events (5.88e-03); Endogenous TLR signaling (2.84e-02); p75(NTR)-mediated signaling (3.18e-02)
KLK6	5653	Alpha-synuclein signaling (1.78e-05)

*Gene symbol**	*Gene ID*	*PID-Pathways and associated P-Values*
LYN	4067	Glypican 1 network (7.49e-06); Alpha-synuclein signaling (1.78e-05); LPA receptor mediated events (5.22e-05); Signaling events mediated by PTP1B (1.89e-04); Signaling events mediated by Stem cell factor receptor (c-Kit) (2.25e-04); Thromboxane A2 receptor signaling (2.67e-04); EPO signaling pathway (4.47e-04); CXCR4-mediated signaling events (6.26e-04); BCR signaling pathway (6.46e-04); Fc-epsilon receptor I signaling in mast cells (3.43e-03); Ephrin B reverse signaling (3.56e-03); Regulation of p38-alpha and p38-beta (3.90e-03); GMCSF-mediated signaling events (5.88e-03); amb2 Integrin signaling (8.35e-03); IL5-mediated signaling events (9.01e-03); IL8- and CXCR1-mediated signaling events (3.44e-02); PDGFR-beta signaling pathway (3.72e-02); EPHA forward signaling (4.51e-02); IL8- and CXCR2-mediated signaling events (4.73e-02); Class I PI3K signaling events (8.09e-02)
PGR	5241	Validated nuclear estrogen receptor alpha network (2.98e-02); Cellular roles of Anthrax toxin (1.88e-01)
VIM	7431	Caspase cascade in apoptosis (1.55e-02); Aurora B signaling (5.66e-02)

* *CDC7, FOXL2, IGFBP7, LCN2 and STC2* do not map to statistically significant pathways and therefore are not listed above.
