# Supplementary material for: Identification of ovarian cancer associated genes using an integrated approach in a Boolean framework
Source: BMC Syst Biol. 2013 Feb 6;7:12. doi: 10.1186/1752-0509-7-12 (PMC3605242; doi:10.1186/1752-0509-7-12)
Supplement: Additional file 5 — High confidence up/down-regulated genes identified in the Boolean framework with their co-expressed neighbors. [file 1752-0509-7-12-S5.pdf]

Identification of ovarian cancer associated genes using an integrated approach in a Boolean framework

by Gaurav Kumar, Edmond J. Breen and Shoba Ranganathan

Additional File 5

High confidence up/down regulated genes identified in the Boolean framework with their co-expressed neighbours.

Gene	Co-expressed neighbours and associated Pearson's correlation coefficient values
AR	PEX6(0.34259), NAB2(0.33005), DCAF16(0.33348), ANKS1A(0.34499), MAP3K12(0.32575), KLHDC8A(0.35409), SCG5(0.32975), ACSS3(0.31902), RIC8B(0.31577), MAP4(0.32548), RPL22(0.29991), BBS1(0.35532), PTN(0.35319), FMOD(0.37627), MXD4(0.32744), RMND1(0.33160), HRH1(0.33426), PODXL(0.37065), IMPDH2(0.40199), SELENBP1(0.30555), PRELP(0.36943), GPM6A(0.33829), TGFB2(0.30515), PALMD(0.38703), UBXN7(0.31892), ZC4H2(0.30764), TOMM20(0.31840), ID4(0.47574), HAO1(0.32069), CLU(0.37295), ADCK3(0.31371), HADH(0.33781), SLC27A6(0.30148), MBOAT2(0.33187), LZTFL1(0.34368), SETD2(0.30252), IQCA1(0.32768), C12orf35(0.30510), NBEAL2(0.30296), NME5(0.32563), D4S234E(0.31353), PFKM(0.32575), ALDH3A2(0.30916), GCK(0.33183), COL4A5(0.36109), IP6K2(0.30064), TSPYL4(0.38465), SRGAP3(0.37083), ARHGEF9(0.31194), ESRRG(0.33173), KCNMB2(0.34821), COBL(0.32455), MFF(0.31766), ITGB4(0.35554), PLXNB1(0.40771), PDCD1(0.29954), BNC2(0.37310), HCG9(0.32069), MYC(0.39253), ZNF248(0.31541), RBFOX2(0.30795), CD200(0.48138), ACOT11(0.31759), ASF1A(0.35745), LOC100128640(0.37311), LDOC1(0.45980), PIP4K2B(0.35770), SALL2(0.31787), FKBP10(0.35078), RCL1(0.30603), GGPS1(0.31736), TIAM2(0.30325), PRKCQ(0.36756), PAIP2B(0.36942), CRIP2(0.33252), METTL21B(0.34346), RPL31(0.35353)
BUB1	PRPF40A(0.52032), RFC3(0.52688), RBBP4(0.58611), ILF2(0.50906), DONSON(0.55703), CENPA(0.61397), CDK1(0.56811), PBK(0.56338), PEX13(0.50100), DEK(0.52283), NCAPH(0.64468), GINS1(0.51139), MTF2(0.51136), CHEK1(0.64970), PRPF4B(0.51711), MELK(0.51431), VAMP3(0.50533), ATAD2(0.61071), KIF11(0.61674), SKP2(0.50890), DLAT(0.53305), CEP55(0.51101), CLIC4(0.57652), CCNF(0.58988), ZWILCH(0.59923), TMEM48(0.62117), RANBP9(0.52099), KPNA2(0.51550), EML4(0.52165), RACGAP1(0.55445), KIF18A(0.51806), CCT2(0.54614), TMEM194A(0.56692), MAT2A(0.50527), TPX2(0.62785), TSN(0.54119), GPSM2(0.69594), SMC4(0.60988), ERCC6L(0.51417), CEP76(0.50940), CBX3(0.51503), CAPRIN1(0.52328), WHSC1(0.52228), PLK1(0.52188), ARFGEF2(0.51039), CCNB1(0.54080), NRAS(0.59965), CCDC99(0.51380), SMC2(0.58621), PTPRA(0.50879), ASF1B(0.52426), MCM10(0.62360), PDK1(0.52783), TOP2A(0.51034),

Gene	Co-expressed neighbours and associated Pearson's correlation coefficient values
BUB1	CENPF(0.50579), KIAA0101(0.51890), NUSAP1(0.50299), FEN1(0.58413), NIPBL(0.50649), MSH2(0.55246), SNX4(0.51434), KIF14(0.56378), CENPE(0.51296), ASPM(0.67652), BUB1B(0.60490), SELT(0.57939), UBE2S(0.56717), DHFR(0.53239), MAD2L1(0.52057), H2AFZ(0.52368), PRUNE(0.50098), PLK4(0.49902), RRM1(0.53933), MAPRE1(0.50223), OIP5(0.54480), CCNA2(0.64288), DLGAP5(0.56249), ECT2(0.50996), CCNB2(0.55317), MSH6(0.54072), C6orf106(0.51932), DEPDC1(0.55711), KIF23(0.57987), NCAPG2(0.52940), RAD51AP1(0.53265), CDCA8(0.57054), CDV3(0.58213)
CDC7	CDKN3(0.58167), POLQ(0.53074), CENPA(0.62024), CDK1(0.56773), CDCA3(0.57945), DNA2(0.61516), NCAPH(0.59694), GINS1(0.64537), MCM6(0.62419), CHEK1(0.53273), MELK(0.53100), PSRC1(0.55470), KIF11(0.57624), MKI67(0.58336), MCM2(0.54824), DLEU2(0.50068), BARD1(0.50586), DTL(0.52194), RACGAP1(0.64667), TPX2(0.63370), PTTG1(0.53955), UNG(0.53863), NEK2(0.53222), GPSM2(0.52225), KIF15(0.60950), USP1(0.54266), KIF18B(0.63999), KIF2C(0.58756), HMGB2(0.57285), KIF20A(0.55174), ZWINT(0.61077), MLF1IP(0.50557), CCNB1(0.55325), AURKA(0.57052), CKAP5(0.52367), ASF1B(0.61668), TOP2A(0.57618), CENPF(0.58888), KIAA0101(0.57949), FBXO5(0.49977), NUSAP1(0.55231), UBE2C(0.62282), KIF14(0.57276), CIT(0.54014), KNTC1(0.51611), ASPM(0.57590), BUB1B(0.58020), LMNB1(0.52789), FANCI(0.50181), RFC4(0.53572), C13orf34(0.55598), EZH2(0.60012), KIF4A(0.51712), TTK(0.57469), CCNB2(0.60609), ECT2(0.53682), PTCD3(0.52144), CCNE2(0.66249), DEPDC1(0.54079), POLE2(0.53830), HJURP(0.54536), RAD51AP1(0.64453), PRC1(0.61225), PRIM1(0.62636)
CHEK1	RFC3(0.52976), RRM2(0.53851), BUB1(0.64970), TMPO(0.50867), PLK1(0.62184), CCNB1(0.53574), CENPA(0.53993), CDC7(0.53273), NRAS(0.51263), SMC2(0.57959), MCM10(0.54953), CDCA3(0.53745), CENPF(0.50039), DEK(0.58720), FBXO5(0.49936), NCAPH(0.61632), GINS1(0.50025), KIAA0101(0.58875), MCM3(0.52501), CAND1(0.51291), FEN1(0.53470), MSH2(0.53261), NCAPD3(0.53597), KIF14(0.53894), MCM6(0.56430), ASPM(0.59121), BUB1B(0.59860), ATAD2(0.57119), UBE2S(0.52692), LMNB1(0.57764), KIF11(0.66110), MCM2(0.58219), DPM1(0.52146), CCNF(0.58042), ZWILCH(0.52759), TMEM48(0.51757), OIP5(0.56753), DLGAP5(0.56813), KIF4A(0.50549), ECT2(0.53711), GMNN(0.61337), CCNB2(0.57222), CCT2(0.53002), MSH6(0.50973), NUP153(0.50352), TPX2(0.58761), KIF23(0.50296), NCAPG2(0.50427), HJURP(0.54030), RAD51AP1(0.57472), GPSM2(0.59001), PRC1(0.50324), USP1(0.53807), SMC4(0.50222), CDCA8(0.52630)
CHEK2	CEP55(0.52758), ASPM(0.52585)
CLU	SRGAP3(0.50947), SCARA3(0.51987)
DAB2	RAB31(0.54011), CREB3L1(0.58136), IL7R(0.51339), OLFML3(0.51030), MMP2(0.51147), SFRP4(0.49972), SPARC(0.59755), SYNE1(0.59241), CLEC7A(0.52570), DSE(0.56954), FLI1(0.54743), HNMT(0.57281), ACTA2(0.55884), PAPSS2(0.64461), CLEC2B(0.61504), PDGFRA(0.63113), DCN(0.55486), LCP2(0.52393), GLT8D2(0.55228), IL10RA(0.53850), NID2(0.54349), LY96(0.71743), PALLD(0.52002), OLFML1(0.61942), IFFO1(0.57131), LAMB1(0.51364), FGL2(0.51213), DOCK10(0.56392),

Gene	Co-expressed neighbours and associated Pearson's correlation coefficient values
DAB2	IGFBP4(0.63395), ISLR(0.50753), VCAM1(0.59200), ADCY7(0.50814), SAMSN1(0.50799), C1S(0.54527), C1QB(0.51455), EMILIN1(0.52341), HTRA1(0.57476), LIMA1(0.58426), CD69(0.49975), VGLL3(0.56399), CXCL12(0.56296), LHFP(0.61421), LPAR6(0.54527), ITGB2(0.51347), FCGR2A(0.54140), FOLR2(0.52864), MAN1A1(0.53995), CD302(0.57057), NID1(0.54796), ARHGAP15(0.52787), PDZRN3(0.49968), PMP22(0.61583), CD3D(0.52021), ARHGEF6(0.53103), AP3S1(0.51029), MS4A4A(0.52512), TYROBP(0.52827), COLEC12(0.51173), EHD2(0.59848), EVI2B(0.56013), ZEB1(0.51352), ADRA2A(0.53992), RASSF2(0.56601), GIMAP6(0.50063), FLRT2(0.60085), GALNT10(0.50219), ECM2(0.60587), COL16A1(0.52986), C17orf91(0.52958), SLC31A2(0.50144), EMP3(0.53809), EGR2(0.51860), LDB2(0.60648), IRF8(0.53944), EDNRA(0.50436), DOCK4(0.54315), DRAM1(0.59915), C14orf139(0.54800), ZEB2(0.67593), MMP19(0.52348), PTGIS(0.53099), CD4(0.53013), NBL1(0.54283), DOCK2(0.51913), PTPRE(0.56811), FN1(0.56190), CSF1R(0.54982), ACSL1(0.55308), TRAF3IP3(0.54593), S1PR1(0.50474), IGSF6(0.54627), SRPX(0.50813), LY86(0.56112), F13A1(0.61309), SMPDL3A(0.60841), ZCCHC24(0.60678), OLFML2B(0.54909), MN1(0.54318), AEBP1(0.55821), MS4A6A(0.60080), MEF2C(0.52322), FMO1(0.57339), PLS3(0.51768), DPYD(0.57779), OGN(0.50311), FBN1(0.51033), GPR124(0.55363), CRISPLD2(0.51609), C11orf75(0.51866), SLC38A6(0.51094), ZFYVE16(0.50837), TGFBI(0.50263), JAM2(0.51379), TRIM22(0.56451), GYPC(0.54186), FCER1G(0.52460), ASPN(0.50854), SNAI2(0.60530), SERPINF1(0.58582), SPOCK1(0.50253), SPARCL1(0.55169), TGFB1I1(0.58814), CD14(0.55354), THBS2(0.51767), ARHGDIB(0.50014), FILIP1L(0.56697)
FOXL2	PCSK6(0.56623), CADPS(0.54655), BAMBI(0.50028), C7(0.54995), GPRASP1(0.55858), COLEC11(0.61122), GATA4(0.60909), DLK1(0.63617), GATM(0.58267), EDN3(0.53944), STAR(0.62268), TCF21(0.57277), TSPAN8(0.53701)
IGF1R	ITM2C(0.33849), HDGF(0.30240), GPATCH4(0.33026), RHOBTB1(0.30025), RPS17(0.33421), RPS3A(0.31157), NGRN(0.34597), FAM164A(0.31874), ABCA2(0.32731), RPL22(0.30833), EPM2AIP1(0.30027), RPL32(0.30241), RPS9(0.32823), DET1(0.32415), TAF15(0.31973), PPM1H(0.31485), ACVR2B(0.38445), SMO(0.39511), BBS10(0.31137), ZNF292(0.36064), SPIN1(0.42615), CNIH3(0.32465), AEN(0.36556), RPS20(0.34731), RPL10A(0.33563), DHX40(0.31013), CLDN9(0.31147), WWC2(0.31272), CD22(0.33915), C15orf29(0.32413), LRIG1(0.38504), FAM86B1(0.33506), BEX1(0.30357), TMEM43(0.32983), PAM(0.35885), TGIF1(0.31202), DGUOK(0.31867), PITPNC1(0.30393), HIPK1(0.34099), C1orf114(0.29903), MBOAT2(0.36844), KIAA0649(0.33611), EEF2(0.34605), LZTFL1(0.34225), TSNAXIP1(0.36124), ZNF629(0.33310), GGTLC2(0.31455), RBMX(0.37822), SPRY1(0.41181), CCNT1(0.36361), WDR73(0.30693), SEPHS1(0.37063), EEF1B2(0.34007), SH3BP4(0.43292), HNRNPA0(0.32533), VGLL4(0.33106), MEIS1(0.34489), KIF3A(0.31451), FOXJ1(0.37506), RPL15P22(0.32997), HMGA2(0.39995), SEMA3F(0.39102), HHAT(0.33214), ELAVL3(0.33821), IRS1(0.31155), ZNF606(0.30358), FZD10(0.32450), C14orf101(0.33170), ACCN2(0.41208), RPL4(0.36466),

Gene	Co-expressed neighbours and associated Pearson's correlation coefficient values
IGF1R	LOC80054(0.38166), TULP3(0.30239), UNKL(0.33050), ARAP3(0.33285), TFAM(0.30041), FAM189A2(0.31461), MAG(0.35485), CRBN(0.37234), CTF1(0.34419), RPL13(0.31961), NAP1L1(0.31031), ARNT2(0.39565), ZBTB39(0.40408), FAM60A(0.39810), P4HTM(0.34143), AIRE(0.32931), FGF9(0.32174), RPL15(0.32202), EIF1B(0.33166), EIF4B(0.35047), BZRAP1(0.32073), DNALI1(0.33867), PLEKHG6(0.31110), RASL11B(0.31953), LEMD3(0.39453), MPST(0.31022), ZNF423(0.40551)
IRAK1	ARPC5L(0.51700), GSTO1(0.67084), RRAGC(0.51341), RPS17(0.56944), NIPA2(0.56383), NDUFV1(0.54171), NARS(0.59186), AURKAIP1(0.61962), GLT25D1(0.53181), ALDOA(0.56914), NDUFA1(0.56521), DNTTIP2(0.53063), SLC12A9(0.50466), RPL32(0.57201), LDHA(0.50255), CASZ1(0.51760), OPHN1(0.51255), RPL12(0.56281), UBL4A(0.52774), EIF2S1(0.54926), EMD(0.55374), JTB(0.54548), ECHS1(0.53466), ATP6AP1(0.60164), PDHA1(0.51027), RPL39(0.62869), GUSB(0.56289), CFL1(0.56890), BRP44L(0.60035), BAG1(0.55071), RPL37(0.54709), SNRPG(0.56984), NINJ1(0.54346), PLEKHJ1(0.64982), C3orf37(0.53406), CLIC1(0.54330), NDUFS7(0.55431), RPS18(0.50164), RPLP1(0.51982), GLA(0.55405), KRT18(0.51990), HSD17B10(0.57782), RAB1B(0.54162), SLC1A5(0.50202), CDC42SE1(0.50767), KCMF1(0.54104), TTC1(0.51156), TESK1(0.50023), RPS19(0.56008), FBXO7(0.50097), GGCX(0.56401), DDB1(0.50916), UQCR11(0.62189), FAU(0.59980), PIN4(0.51241), ZNF593(0.52857), IPO13(0.52243), CSK(0.62813), PTTG1(0.54034), RPS13(0.51213), RPS29(0.59744), FAM136A(0.57508), UQCRQ(0.54924), THOC7(0.52896), RPL8(0.50731), RPL28(0.54907), ZNF544(0.51416), COX8A(0.52958), TALDO1(0.52363), CARS(0.58178), LRRFIP2(0.50101), G6PD(0.53161), GPX1(0.52790), FTH1P5(0.59425), RPLP0(0.57079), PSME2(0.58390), YARS(0.61392), TBC1D9B(0.55741), ANKRD28(0.50182), COX7B(0.61103), NSDHL(0.54236), EEF1G(0.54084), PSME1(0.51833), UBA52(0.51596), PPA1(0.56173), ATP5J2(0.50867), C20orf24(0.54878), SSR4(0.61238), POLR2H(0.56631), SND1(0.50576), TUBA1B(0.58163), NDUFB2(0.65958), HAX1(0.53505), INTS5(0.56780), BCAP31(0.65374), RPL41(0.54907), RAB13(0.55332), EBP(0.52037), USP3(0.50954), C12orf44(0.58130), CCDC9(0.50148), NAA10(0.76244), RBCK1(0.53881), TUBA1C(0.56711), TAF10(0.51794), FAM53B(0.60174), GDI1(0.51819), LAGE3(0.72114), NDUFC2(0.51119), KIAA0930(0.50097), MEA1(0.57307), PSMD2(0.60590), RPL30(0.51262), GRHPR(0.51301), RAB8A(0.50101), OAZ1(0.65986), ACTG1(0.51734), SLC25A5(0.55301), COX7A2(0.59455), KDM2A(0.57134), ANP32B(0.55514), RPS15(0.54172), ATP5L(0.63424)
KLK6	LYPD1(0.56349), KLK10(0.62749), KLK7(0.69160), KLK8(0.65798), WT1(0.50073), ZBED2(0.52437)
LYN	NFKB1(0.58010), ACOT9(0.50700), SERPINB1(0.52357), TMCO1(0.50079), JOSD1(0.50483), PAPOLA(0.50200), KIAA0368(0.49907), CTSS(0.59194), RAB9A(0.50644), PSMA3(0.50104), STAT3(0.54603), CD59(0.57332), CFLAR(0.54850)
PGR	PIP(0.31536), C11orf16(0.34393), NDP(0.30109), GAD1(0.34337), FUT3(0.30767), ATP8B4(0.31441), TFF3(0.35591), VPS13D(0.32194), SCNN1B(0.32086), GSTA3(0.32416), MB(0.32275), RIMS2(0.32574), DNAH6(0.37492), SLC47A1(0.39339),

Gene	Co-expressed neighbours and associated Pearson's correlation coefficient values
PGR	PTCH2(0.34762), TSPAN8(0.30883), CCNO(0.32182), RAB36(0.38977), DHRS9(0.30331), ALPP(0.31362), MSMB(0.32542), DLEC1(0.32383), DNAH9(0.32173), MPPED2(0.37866), EFCAB1(0.32559), RFK(0.30193), FAM5B(0.33546), KIAA0319(0.33451), RASGRF1(0.31722), IFT88(0.31820), SPAG1(0.32363), RTDR1(0.30214), LRRC50(0.37941), GREB1(0.36624), MAOB(0.31811), TSNAXIP1(0.31117), SYBU(0.32847), NRTN(0.32369), KCNN3(0.29978)
STC2	SHMT2(0.35727), PCK2(0.33136), SLC25A31(0.31435), TRIB3(0.38421), ZNF473(0.30848), FANCA(0.33840), NAA16(0.30505), INTS6(0.32968), ASNS(0.41813), XPO4(0.31964), HOXC10(0.34740), YARS(0.31377), NXPH4(0.32258), P4HA1(0.34997), EIF4EBP1(0.36274), EXOSC8(0.30158), WNT5A(0.33121), DOHH(0.34541), ACVR2A(0.32137), KIAA1466(0.41478), WDR33(0.32975), RAD51L1(0.32323), ENO2(0.33506), ATF4(0.34500), PPFIA4(0.32205), SLC7A5(0.40732), CLK4(0.30427), BNIP3(0.43934), MGAT3(0.32159)
VIM	IL6ST(0.57733), MAP1LC3B(0.53231), FSTL1(0.54740), HEXB(0.53365), CD99(0.51442), ACTB(0.56107), IGFBP4(0.54571), PLAU(0.50550), BGN(0.51439), RHOBTB3(0.53695), CEP170(0.51788), SH3BGRL(0.54848), DSE(0.51349), TSPAN4(0.51155), C19orf10(0.50887), FTL(0.54032), FZD1(0.57494), HTRA1(0.49983), MYO1D(0.54607), IFNGR1(0.56515), SERINC1(0.53565), RASA1(0.50859), DCN(0.51984), RTN4(0.52159), COL3A1(0.54354), PSAP(0.51069), PMP22(0.55380), GABARAP(0.54919), VCAN(0.55220), PALLD(0.57897), MARCKS(0.56104), ILK(0.52793)
