# Supplementary material for: Identification of ovarian cancer associated genes using an integrated approach in a Boolean framework
Source: BMC Syst Biol. 2013 Feb 6;7:12. doi: 10.1186/1752-0509-7-12 (PMC3605242; doi:10.1186/1752-0509-7-12)
Supplement: Additional file 6 — Schematic representation of co-expressed genes with significant Boolean values. Edges are colour-coded to highlight the range of pearson’s correlation coefficient in co-expression network: black (> 0.7), slate grey (0.65 - 0.7), navy blue (0.60 - 0.65), slate blue ( 0.55- 0.60), dark green (0.50 – 0.55), dark olive green (0.45 – 0.50 ). Yellow (0.40 -0.45), indian red (0.35 -0.40 ) and peru (0.30 - 0.35). [file 1752-0509-7-12-S6.pdf]

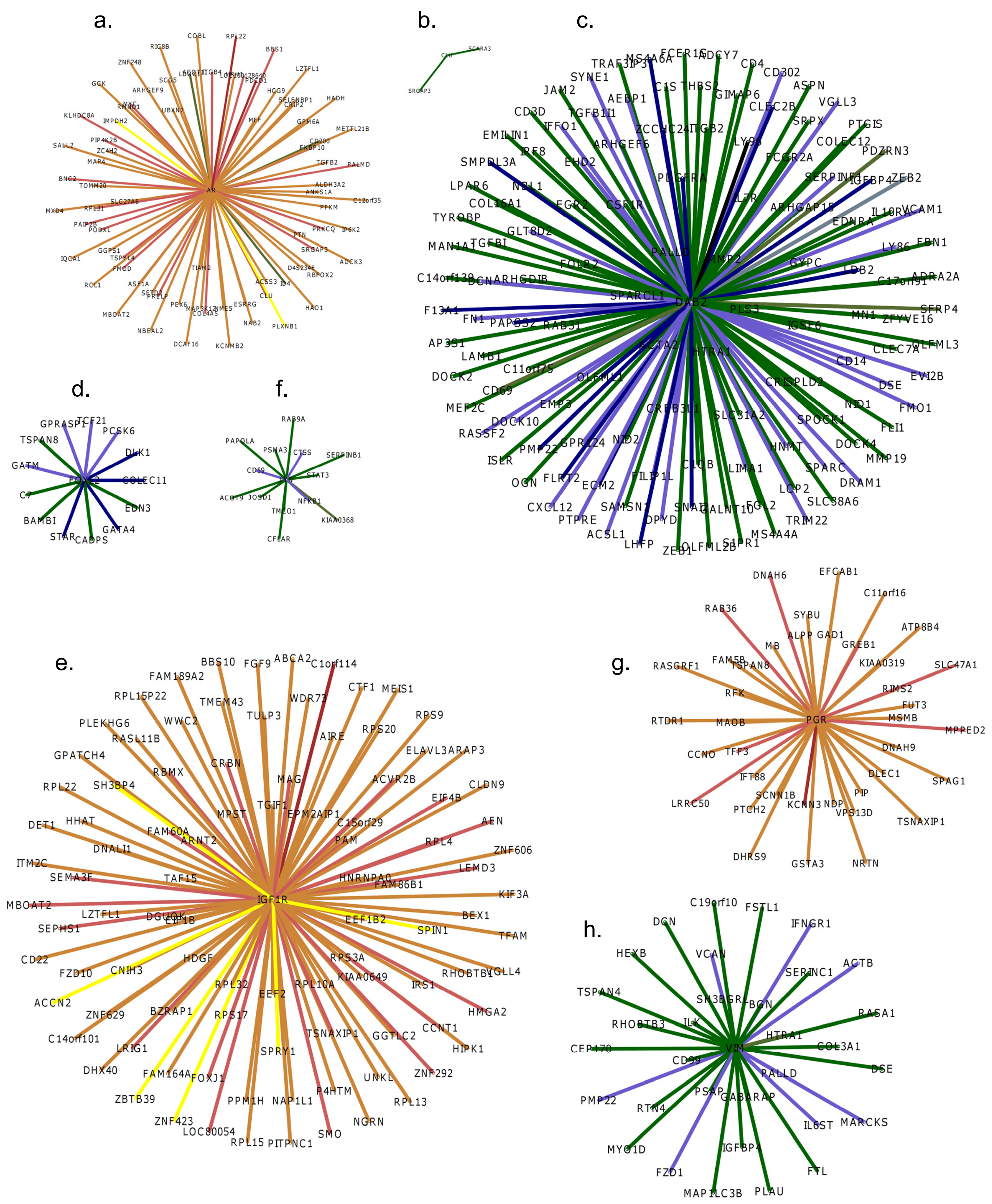

Figure S1: Co-expression of down-regulated and validated genes.
Schematic representation of up-regulated and validated genes: a. *AR*, b. *CLU*, c. *DAB2*, d. *FOXL2*, e. *IGFR1*, f. *LYN*, g. *PGR* and h. *VIM*. Edges are colour-coded to highlight the range of Pearson's correlation coefficient in co-expression network: black (>0.7), grey (0.65-0.7), dark blue (0.60 - 0.65), blue (0.55-0.60), dark green (0.50-0.55), green (0.45-0.05), yellow (0.40-0.45), red (0.35-0.40) and brown (0.30-0.35). Co-expressed neighbours and their associated co-expression Pearson's correlation values are available from Additional File 5.
