# Supplementary material for: Identification of ovarian cancer associated genes using an integrated approach in a Boolean framework
Source: BMC Syst Biol. 2013 Feb 6;7:12. doi: 10.1186/1752-0509-7-12 (PMC3605242; doi:10.1186/1752-0509-7-12)
Supplement: Additional file 7 — GATHER [[66]] GO biological process annotations of the 17 differentially expressed genes associated with the cancer hallmarks in Table3. [file 1752-0509-7-12-S7.pdf]

Identification of ovarian cancer associated genes using an integrated approach in a Boolean framework by Gaurav Kumar, Edmond J. Breen and Shoba Ranganathan

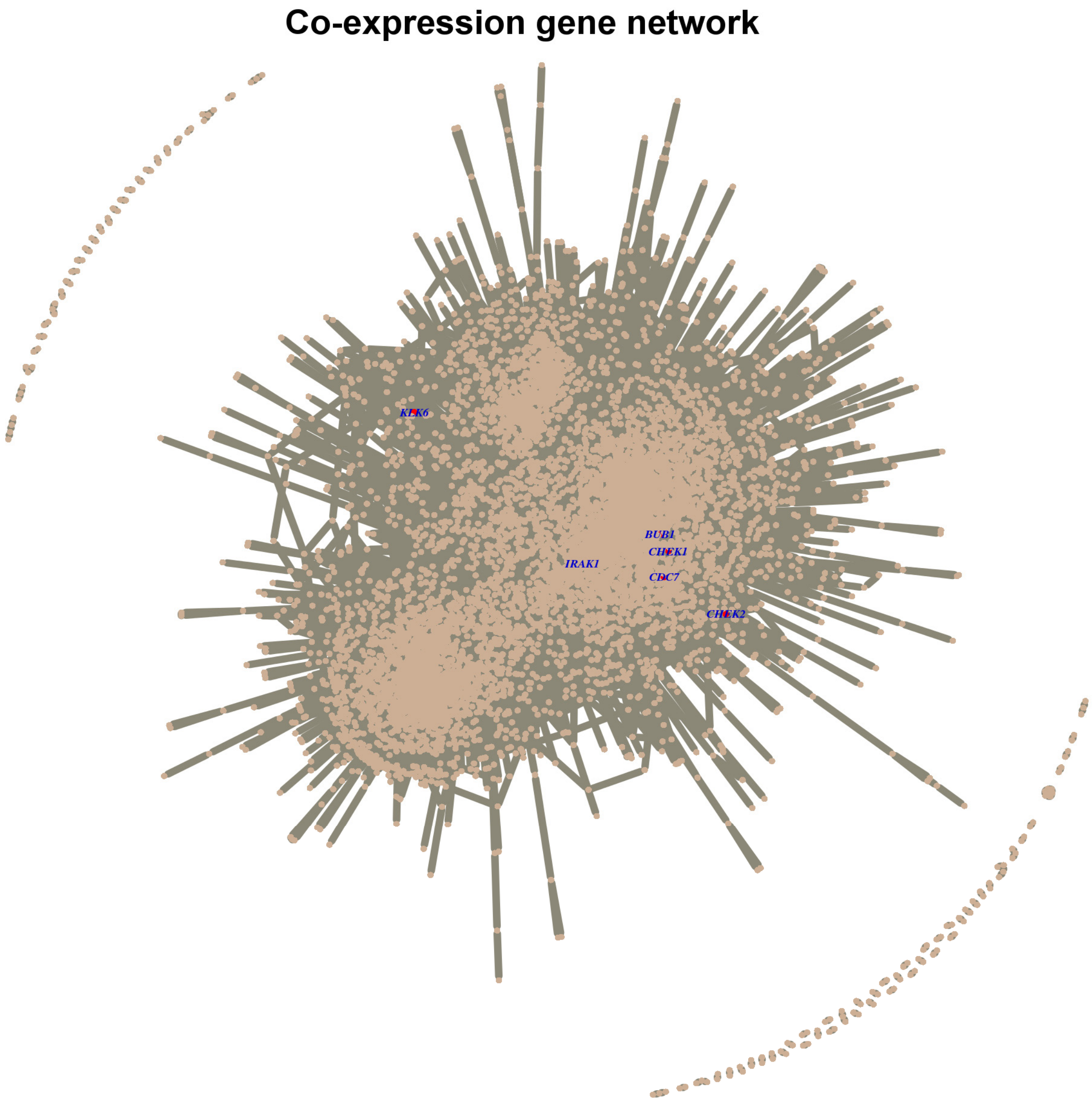

Additional File 7: Co-expression gene network with Pearson's correlation coefficient > 0.5.

Up-regulated genes (*IRAK1*, *CDC7*, *KLK6*, *BUB1, CHEK1*, and *CHEK2*) of the TCGA data are mapped as a co-expression network (GSE3149). *STC2* not represented as the average Pearson's co-correlation coefficient < 0.5
