# Supplementary material for: Identification of ovarian cancer associated genes using an integrated approach in a Boolean framework
Source: BMC Syst Biol. 2013 Feb 6;7:12. doi: 10.1186/1752-0509-7-12 (PMC3605242; doi:10.1186/1752-0509-7-12)
Supplement: Additional file 8 — Relevant GO biological process characterization from GeneCards [[67]] for the 17 differentially expressed genes, mapped to cancer hallmarks (HM) in Table 3. [file 1752-0509-7-12-S8.pdf]

Identification of ovarian cancer associated genes using an integrated approach in a Boolean framework
by Gaurav Kumar, Edmond J. Breen and Shoba Ranganathan

Additional File 8: GATHER [71] GO biological process annotations of the differentially expressed genes associated with the cancer hallmarks in Table 3.

Genes are annotated with GO terms using the GATHER enrichment method. B-factor (i.e. Bayes factor) quantifies the amount of evidence supporting the hypothesis that the annotation is associated with our genes. *p-values* and *q-values* are based on Fisher's exact test.

Cancer Hallmark	*GO Term Definition*	*GO Term-ID (GO-depth)*	*Genes*	*ln(B-factor)*	*p-value*	*q-value*
1. Sustain growth signal	negative regulation of cell proliferation	GO:0008285 (6)	CDC7; CHEK1; IGFBP7	4.38	0.000724521	0.00937227
2. Escape growth suppressor i.e. Insensitivity to growth inhibitor	regulation of cell proliferation	GO:0042127 (5)	CDC7; CHEK1; IGF1R; IGFBP7	5.05	0.00037447	0.006219909
3. Active invasion and metastasis	negative regulation of programmed cell death	GO:0043069 (6)	IGF1R	-0.82	0.135335283	0.227637688
4. Limitless replicative potential / Enable replicative immortality	cell proliferation	GO:0008283 (4)	AR; BUB1; CDC7; CHEK1; CHEK2; DAB2; IGF1R; IGFBP7	7.45	3.53575E-05	0.001294022
5. Induces angiogenesis	tissue regeneration	GO:0042246 (4)	KLK6	2.6	0.004127844	0.03337327
6. Resisting cell death	anti-apoptosis	GO:0006916 (8)	IGF1R	-0.71	0.120031629	0.220909978
7. Genomic instability	DNA damage checkpoint	GO:0000077 (7)	CHEK1; CHEK2	6.54	8.02792E-05	0.001949856
8. Deregulate cellular energetics	regulation of cellular physiological process	GO:0051244 (4)	CDC7; CHEK1; IGF1R; IGFBP7	2.26	0.006604527	0.039955058
9. Avoid immune destruction						
10. Tumor promoting inflammation	wound healing	GO:0042060 (6)	KLK6	2.09	0.006874063	0.040356613
