# Supplementary material for: Identification of ovarian cancer associated genes using an integrated approach in a Boolean framework
Source: BMC Syst Biol. 2013 Feb 6;7:12. doi: 10.1186/1752-0509-7-12 (PMC3605242; doi:10.1186/1752-0509-7-12)
Supplement: Additional file 9 — Gene expression data for the 17 genes identified in this study across 45 (38 tumor + 7 normal) samples. [file 1752-0509-7-12-S9.pdf]

Identification of ovarian cancer associated genes using an integrated approach in a Boolean framework by Gaurav Kumar, Edmond J. Breen and Shoba Ranganathan

Additional File 9: Relevant GO biological process characterization from GeneCards [72] for the 17 differentially expressed genes, mapped to cancer hallmarks (HM) in Table 3.

Gene symbol	*GO ID*	*GO term {HM}*	*Evidence**	*PubMed IDs*
KLK6	GO:0009611	response to wounding {10}	NAS	12016211
	GO:0042246	tissue regeneration {5}	NAS	12878203
	GO:0045595	regulation of cell differentiation {2}	NAS	11668196
IRAK1	GO:0031663	8. lipopolysaccharide-mediated signaling pathway {8}	IMP	10383454
	GO:0032088	negative regulation of NF-kappaB transcription factor activity {5}	IMP	10383454
	GO:0045893	positive regulation of transcription, DNA-dependent {4}	NAS	11397809
	GO:0008284	positive regulation of cell proliferation {2}	IMP	17102137
	GO:0070498	interleukin-1-mediated signaling pathway {10}	IMP	10383454
CDC7	GO:0000075	cell cycle checkpoint {7}	TAS	--
	GO:0006260	DNA replication {4}	TAS	--
	GO:0008284	positive regulation of cell proliferation {2}	IMP	17102137
	GO:0033261	regulation of S phase {7}	IMP	17102137
CHEK1	GO:0000077	DNA damage checkpoint {7}	IMP	19716789
	GO:0006260	DNA replication {7}	TAS	--
	GO:0006281	DNA repair {7}	IMP	19716789
	GO:0010569	regulation of double-strand break repair via homologous recombination {7}	IDA	15665856
BUB1	GO:0007093	mitotic cell cycle checkpoint {7}	TAS	9521327
	GO:0007094	mitotic cell cycle spindle assembly checkpoint {7}	TAS	9790499
CHEK2	GO:0000077	DNA damage checkpoint {7}	TAS	10617473
	GO:0006302	double-strand break repair {7}	IMP	18317453
	GO:0006355	regulation of transcription, DNA-dependent {4}	IDA	12717439
	GO:0006974	response to DNA damage stimulus {7}	TAS	9836640
	GO:0008630	DNA damage response, signal transduction resulting in induction of apoptosis {7}	IMP	12717439
	GO:0045893	positive regulation of transcription, DNA-dependent {4}	IDA	17101782
STC2	GO:0007166	cell surface receptor signaling pathway {2}	TAS	9753616
	GO:0007267	cell-cell signaling {1}	TAS	9753616
	GO:0008283	cell proliferation {4}	TAS	23187001**
	GO:0016477	cell migration {3}	TAS	23187001**

Gene symbol	*GO ID*	*GO term {HM}*	*Evidence**	*PubMed IDs*
DAB2	GO:0008283	cell proliferation {4}	TAS	9620555
	GO:0032436	positive regulation of proteasomal ubiquitin-dependent protein catabolic process {8}	IMP	12805222
	GO:0045892	negative regulation of transcription, DNA-dependent {5}	IMP	12805222
	GO:0045893	positive regulation of transcription, DNA-dependent {4}	IMP	12805222
	GO:0090090	negative regulation of canonical Wnt receptor signaling pathway {1}	IMP	12805222
VIM	GO:0006915	apoptotic process {6}	TAS	--
	GO:0006921	cellular component disassembly involved in apoptotic process {6}	TAS	--
	GO:0006928	cellular component movement {3}	TAS	16130169
FOXL2	GO:0006309	apoptotic DNA fragmentation {7}	IMP	16153597
	GO:0030154	cell differentiation {4}	NAS	12471206
	GO:0043065	positive regulation of apoptotic process {6}	IMP	16153597
	GO:0043280	positive regulation of cysteine-type endopeptidase activity involved in apoptotic process {6}	IMP	16153597
	GO:0045892	negative regulation of transcription, DNA-dependent {5}	IDA	19744555
LCN2	GO:0043069	negative regulation of programmed cell death {3}	TAS	23056397**
	GO:0006916	anti-apoptosis {6}	TAS	23056397**
PGR	GO:0007267	cell-cell signaling {1}	TAS	1557371
	GO:0007165	signal transduction {1}	TAS	1557371
AR	GO:0008283	cell proliferation {4}	NAS	10075738
	GO:0008284	positive regulation of cell proliferation {2}	IDA	17277772
	GO:0016049	cell growth {1}	NAS	15572661
IGF1R	GO:0006916	anti-apoptosis {6}	TAS	8710868
	GO:0006955	immune response {9}	IMP	16886151
	GO:0008284	positive regulation of cell proliferation {2}	TAS	10749889
	GO:0030335	positive regulation of cell migration {3}	IMP	12138094
	GO:0043066	negative regulation of apoptotic process {3}	IDA	12556535
	GO:0045740	positive regulation of DNA replication {4}	IMP	12138094

Gene symbol	*GO ID*	*GO term {HM}*	*Evidence**	*PubMed IDs*
LYN	GO:0002513	tolerance induction to self antigen {9}	TAS	15489917
	GO:0002768	immune response-regulating cell surface receptor signaling pathway {9}	TAS	15489917
	GO:0006974	response to DNA damage stimulus {7}	IDA	10891478
	GO:0008285	negative regulation of cell proliferation {1}	IMP	14726379
	GO:0033628	regulation of cell adhesion mediated by integrin {4}	IMP	18802065
	GO:0042981	regulation of apoptotic process {3/6}	IDA	11517336
	GO:0050777	negative regulation of immune response {9}	TAS	15489917
	GO:0051272	positive regulation of cellular component movement {3}	IDA	16467205
	GO:0070668	positive regulation of mast cell proliferation {3}	IMP	11435302
IGFBP7	GO:0007155	cell adhesion {4}	IDA	8117260
	GO:0008285	negative regulation of cell proliferation {1}	TAS	8939990
CLU	GO:0006956	complement activation {9}	TAS	1585460
	GO:0032463	negative regulation of protein homooligomerization {4}	IMP	16113678
	GO:0043066	negative regulation of apoptotic process {3}	IMP	16113678

* **Experimental Evidence Codes:**

IDA: Inferred from Direct Assay

IMP: Inferred from Mutant Phenotype

Author Statement Evidence Codes:

TAS: Traceable Author Statement

** Literature search by authors and not currently in GeneCards
